# Citrus Essential Oils in Aromatherapy: Therapeutic Effects and Mechanisms

**DOI:** 10.3390/antiox11122374

**Published:** 2022-11-30

**Authors:** Pooja Agarwal, Zahra Sebghatollahi, Mehnaz Kamal, Archana Dhyani, Alpana Shrivastava, Kiran Kumari Singh, Mukty Sinha, Neelima Mahato, Awdhesh Kumar Mishra, Kwang-Hyun Baek

**Affiliations:** 1 Division of Chemistry, School of Basic and Applied Sciences, Galgotias University, Greater Noida 203 201, Uttar Pradesh, India; 2 Department of Plant Breeding and Biotechnology, Faculty of Agricultural Sciences and Food Industries, Science and Research Branch, Islamic Azad University, Tehran 1477893855, Iran; 3 Department of Pharmaceutical Chemistry, College of Pharmacy, Prince Sattam Bin Abdulaziz University, Al Kharj 11942, Saudi Arabia; 4 Department of Applied Sciences, School of Engineering, University of Petroleum and Energy Studies, Dehradun 248 007, Uttarakhand, India; 5 Department of Botany, Sri Shankar College, (A Constituent Unit of V.K.S.U., Ara), Rohtas, Sasaram 821 115, Bihar, India; 6 Department of Geography, Central University of South Bihar, Gaya 151001, Bihar, India; 7 Department of Medical Devices, National Institute of Pharmaceutical Education and Research, Ahmedabad 382 355, Gandhinagar, India; 8 School of Chemical Engineering, Yeungnam University, Gyeongsan 38541, Gyeongsangbuk-do, Republic of Korea; 9 Department of Biotechnology, Yeungnam University, Gyeongsan 38541, Gyeongsangbuk-do, Republic of Korea

**Keywords:** citrus essential oils, aromatherapy, natural aromatic compounds, therapeutic effects of citrus EOs, characterization of citrus EOs

## Abstract

Citrus is one of the main fruit crops cultivated in tropical and subtropical regions worldwide. Approximately half (40–47%) of the fruit mass is inedible and discarded as waste after processing, which causes pollution to the environment. Essential oils (EOs) are aromatic compounds found in significant quantities in oil sacs or oil glands present in the leaves, flowers, and fruit peels (mainly the flavedo part). Citrus EO is a complex mixture of ~400 compounds and has been found to be useful in aromatic infusions for personal health care, perfumes, pharmaceuticals, color enhancers in foods and beverages, and aromatherapy. The citrus EOs possess a pleasant scent, and impart relaxing, calming, mood-uplifting, and cheer-enhancing effects. In aromatherapy, it is applied either in message oils or in diffusion sprays for homes and vehicle sittings. The diffusion creates a fresh feeling and enhances relaxation from stress and anxiety and helps uplifting mood and boosting emotional and physical energy. This review presents a comprehensive outlook on the composition, properties, characterization, and mechanism of action of the citrus EOs in various health-related issues, with a focus on its antioxidant properties.

## 1. Introduction

Citrus is one of the world’s most abundant fruits containing substantial amounts of beneficial secondary metabolites [1]. Among them, citrus essential oils (EOs) are important secondary metabolites; they are usually aromatic compounds found in oil glands present in the flowers, leaves, and fruit peels. However, most citrus EOs are extracted from fruit peels, viz., fruit rind, or flavedo (green part) and albedo (white part). These citrus EOs contain 85–99% volatile and 1–15% non-volatile components [2] and their content as well as chemical composition depend on species and extraction methods [3,4,5]. These volatile constituents contain large amounts of monoterpene hydrocarbons (70–95%) and *d*-limonene, a good source of antioxidants, in all the reported orange species [6].

Aromatherapy using citrus EOs has been practiced as a treatment method since ancient times. Aromatherapy is utilized to relieve many symptoms, such as body pain, nausea, vomiting, anxiety, depression, stress, insomnia, etc. [7]. Several scientific reports have been published regarding the use of EOs in the treatment of a number of medical issues, including hypertension, hypotension, cognitive dysfunction [8,9,10,11,12], physical and psychological stress, and exhaustion [13]. EOs are extracted from plants and used in a controlled manner in aromatherapy with few or no side effects. Currently, EOs are hugely popular as safe and natural agents with medicinal and therapeutic properties and have been approved by the US Food and Drug Administration (FDA).

Citrus EOs are fragrant volatile molecules, which upon inhalation can alter hemodynamic parameters or blood flow in the body by controlling circulation through the autonomous nervous system. Citrus EOs have also been investigated for their antimicrobial [14] and antioxidant activities [15,16]. Many citrus EOs, such as orange [17] and bitter orange [18,19] have shown anxiolytic, antidepressant, anticonvulsant, analgesic, and sedative effects and influence overall emotional behavior. Major components in the citrus EOs include bioactive compounds, such as monoterpenes and its derivatives, aldehydes, ketones, esters, alcohols, limonene, *β*-pinene, and *γ*-terpinene [20]. 

### Global Production and Consumption of Citrus 

Natural products are popular globally due to their nutritional value and little or no side effects. The demand for citrus EOs has been continuously increasing to produce higher quality nutraceuticals, food and beverages, bakery, natural preservatives for vegetables, meat and fish, pharmaceuticals, aromatherapy, perfumes, toiletries, and personal care, blending ingredients for herbal tea, cosmetic ingredients, and so on [21]. The major citrus-producing countries, climate sustainability, and annual production of the different citrus fruits in different geographical regions are shown in Figure S1 (Supplementary Materials).

**Figure 1:**
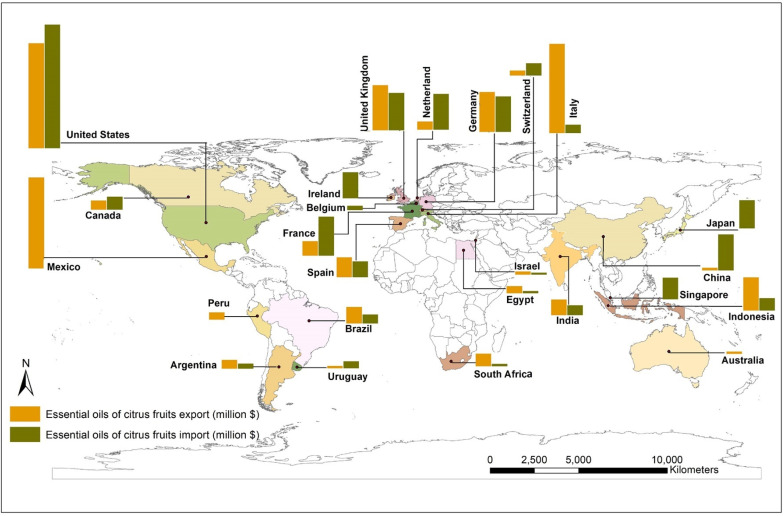
Market of essential oils obtained from citrus fruits, (2020) (Note: Bar graph was created for major countries involved in import and export; the map was created using ArcGIS 10.8.1 with UTM projection taking WGS84 datum) [24].

The global market of citrus EOs in the year 2018 was 6.31 billion USD, which is predicted to grow at a rate of 6.5% by the year 2025. The market has been estimated to grow up to 9.43 billion by the year 2028 [22,23]. The market segregation of citrus essential oils and the global market for citrus EOs based on its major applications (in %; up to the year 2018) are shown in Figure S2 (Supplementary Materials). Global citrus oil market by application (by the year 2018) [22,24] and citrus EO market value forecast [25] are shown in Figure S3a,b (Supplementary Materials). The market of EOs obtained from citrus fruits (data year 2020) and its market value forecast for the decade are displayed in the world map in Figure 1. Many countries in the Asia-Pacific region have a high demand for citrus EOs because of their use in various food and beverages, cosmetic preparations, and therapeutics. Similarly, the demand is expected to increase in Europe and the US due to their higher usage in the food and beverage industry and the substantial use of these products in aromatherapies. In addition, citrus EOs are also becoming a preferred ingredient material in green repellents and pesticides against insects and pests [26].

## 2. Extraction, Characterization, and Authentication Methods for Citrus EOs

EOs are present in the oil glands in the peel and cuticles of the citrus fruit peel or pericarp. The EOs are released when oil sacs are crushed during juice extraction or under pressure during cold pressing of the peel waste. The major component of the EOs is *d*-limonene [14,20]. Cold pressing has been a traditional method of extracting essential oils and the yield is a watery emulsion. The latter is centrifuged to recover the EOs [27]. Alternately, the extraction of EOs is also carried out using stream stripping and distillation methods. These methods have been found effective and efficient in removing oil components from oil-milled sludge. Modern methods include distillation techniques, e.g., Microwave Steam Distillation (MSD), Microwave Hydrodiffusion and Gravity (MHG), and Instant Controlled Pressure Drop Technique (DIC). Instant controlled pressure drop (DIC) technology facilitates the extraction of essential oil as well as the expansion of the plant matrix. This improves the extraction of the oil significantly. In this method, a high-steam pressure (~0.6–1.0 MPa) is applied constantly for a short time (~5–60 s) followed by an instantaneous drop in the pressure towards vacuum 5 kPa with a rate ≥0.5 MPa s^−1^. This treatment results in the rapid expansion of the sample matrix, auto evaporation, and faster cooling, enabling the extraction of volatile compounds and EOs within 1–4 min. The EOs obtained by distillation have been observed to deteriorate easily and develop off-odor because of the instability of the terpene hydrocarbons, e.g., *d*-limonene [28]. Supercritical fluid extraction (SFE) is an emerging and inexpensive technique of extraction and isolation of EOs [29]. By this method, efficient and fast extraction can be done at ambient temperatures, without incorporating clean-up steps in absence of harmful organic solvents. Carbon dioxide (CO_2_) is used in the SFE method because of its non-explosive, non-toxic nature along with the ease availability. CO_2_ can be considered an ideal solvent and can be easily eliminated from extracted products [30,31].

MHG is a highly efficient method as it accelerates the extraction process many times over. In addition, it also enables the recovery of EOs without any changes in the oil composition. MSD techniques have an edge over MSD as it causes more rapid rupture of the cell wall of the plant material under strong microwaves which quickly releases the cell cytoplasm containing oils. The main extraction methods/techniques for obtaining citrus EOs are summarized in Table S1 (Supplementary Materials) [4].

The extraction process yields a matrix containing a mixture of phytochemicals which ought to undergo separation, purification, and isolation to obtain individual compounds. Citrus EO is a complex mixture of ~400 volatile and semi-volatile compounds. Column chromatography, high-speed counter current chromatography (HSCC), and high-performance liquid chromatography (HPLC) are generally employed involving solvent combinations, such as hexane:n-butanol, ethyl acetate:hexane, butanol:water, chloroform:methanol, etc., in the basic process of purification and separation of compounds. The different compounds are detected and quantitatively determined using a combination of modern instruments, viz., UV–visible, mass spectroscopy, and HPLC. GC and its extensions such as MDGC, enantioselective capillary gas chromatography (eCGC), ultra-high performance liquid chromatography, etc. are the most extensively employed for EO separation, identification, and quantitative characterization due to its volatility and complexity of most natural oils [32].

Gas chromatography (GC) is one of the most popular methods for the characterization of single-phase vapor samples and is suitable for samples with 2 (molecular hydrogen) to 1500 mass units. Almost all EOs fall within this mass range. A combination of GC and MDGC with other techniques, such as mass spectroscopy (MS) and Raman spectroscopy is employed to improve the efficiency of the separating power of chromatography and analyze more complex structures. Such coupled analyses improve data quality and exhibit good separation performance [33]. The use of two or more techniques detects adulteration in EOs extracted from the citrus peels and waste more accurately [34]. Researchers used simultaneous distillation and extraction (SDE)-GC-MS and MDGC-MS techniques to study and authenticate the enantiomeric ratios of chiral compounds present in the citrus EOs. These techniques helped in the identification of 67 volatile compounds, including limonene, γ-terpinene, and linalool, as the major compounds and sabinene, camphene, and *β*-phellandrene as minor and chiral aromatic components in lemon and lime. A combination of MDGC and GCC-IRMS is employed to determine the authenticity of the EOs extracted from neroli (Egyptian bitter orange flower) and lime [35]. A comparative analysis was performed for lime (*Citrus aurantifolia* Swingle and *Citrus latifolia* Tanaka)-based Eos following two different approaches using MDGC and gas chromatography–combustion-isotope ratio mass spectrometry (GC–C-IRMS). This study is the first to differentiate Eos extracted from Persian lime and key lime. A series of components were identified including limonene, geranial, *β*-caryophyllene, trans-*α*-bergamotene, *α* and *β*-pinene, and germacrene B, using GC–C-IRMS. MDGC determined the enantiomeric distribution of camphene, limonene, linalool, *α*-phellandrene, *β*-phellandrene, *β*-pinene, terpinen-4-ol, *α*-terpineol, sabinene, and α-thujene in lime oils. Such hyphenated techniques are also used successfully in the investigation of citrus oil-based flavored drinks. Italian alcoholic syrup was examined by comparing carbon isotope ratios to identify the presence of genuine cold-pressed peel oils. For this purpose, solid phase microextraction was performed, followed by GC with IRMS. GC was used to determine the enantiomeric distribution of the selected volatile chiral samples, whereas qualitative analyses of the samples were performed by mass spectrometry. The results were confirmed using enantioselective gas chromatography [36].

Ultra-high-performance liquid chromatography–time-of-flight–mass spectrometry (UHPLC–TOF–MS) profiling and ^1^H nuclear magnetic resonance (NMR) near-infrared spectroscopy are employed for the fingerprinting of lemon oil [37]. Metabolite variations have been also investigated in lemon oil samples. Such analysis has high demand in the fragrance and flavor industries for terpenoids, citropten, bergamottin, furocoumarins, flavonoids, and fatty acids. Characterization based on quantitative analysis of substances present in EOs is an important process in essential oil-based industries. The different methods/techniques of characterization/authentication of citrus EOs have been summarized in Table S2 (Supplementary Materials).

## 3. Components of Citrus EOs 

Citrus species are rich in various EOs, with many chemical components of interest for aromatherapy. Several ingredients used in pharmaceuticals and cosmetics are procured from citrus EOs [38,39,40,41]. Around 400 compounds, which cover 85–99% of the total oil fraction, have volatile and semi-volatile properties [38,39,42,43]. Citrus EOs can be grouped into five major classes: hydrocarbon monoterpenes, oxygenated monoterpenes, hydrocarbon sesquiterpenes, and oxygenated sesquiterpenes. The major component of citrus Eos is limonene, which can be found in quantities ranging from 32% to 98% [44]. Hydrocarbons, aliphatic aldehydes, and oxygen-containing mono- and sesquiterpenes are the next most significant classes of compounds present in citrus EOs which show antioxidant properties. Several terpenes are present as their functionalized derivatives, which are volatile compounds, and flavonoids, diterpenoids, sterols, coumarins, and fatty acids are some of the non-volatile compounds [45]. Several studies have reported the chemical composition of EOs derived from the citrus flower, leaf, and peel. The composition of citrus EOs varies with citrus species, origin, climatic and geographical conditions, ripening, method of extraction, etc. [14]. The molecular structures of the volatile and non-volatile compounds present in citrus EOs are displayed in Figure S4 (Supplementary Materials). The composition of the aromatherapeutic components present in the EOs of common citrus species [3,14] are summarized in Figure S5 (Supplementary Materials).

## 4. Aromatherapy: Mechanisms 

### 4.1. Evolution of Aromatherapy

Stress conditions alter the respiration process, and an altered respiration activates the limbic system (amygdala, hippocampus, and hypothalamus) in the brain and induces psycho-physiological responses. The latter can alter the emotional responses. This is how respiration relates to emotional behavior and brain functions. Furthermore, pulmonary diseases have been observed to affect brain-cell growth, reduce oxygen supply in the body and brain causing neurophysiological and neurobehavioral disorders, namely anxiety and depression. Moreover, the systemic circulation carrying blood with insufficient oxygen supply also transports lung-induced inflammation mediators. The latter induces adaptive responses in the brain and the body. Applications of EOs have been observed to impart neuroprotective and anti-aging effects, and relief from respiratory congestion, pain, insomnia, anxiety, depression, stress, and other psychological and physiological disorders mostly due to its antioxidant properties [46]. When inhaled, the EOs can stimulate the olfactory, respiratory, and gastrointestinal systems, and the EOs release endorphins to initiate a feeling of well-being and an analgesic effect [7]. Citrus EOs have been reported to be safe and effective for treating insomnia. Moreover, these can decrease the side effects of drugs and sleep illnesses owing to their short- or long-term usage [47]. The EOs have gained attention in clinical and scientific research because they are harmless and do not have any side effects [46]. There are three ways by which EOs can reach and act directly on the respiratory, circulatory, and central nervous systems, viz., (i) inhalation through the respiratory tract; (ii) oral intake in the form of capsules, drops, or food; and (iii) topical absorption through the skin [48]. 

### 4.2. Mechanism

#### 4.2.1. Inhalation

A human can differentiate more than 10,000 types of aromas. Humans possess ~400 functional gene coding for olfactory sensory neurons (OSNs). Each receptor expresses a specific type of odorant reception [49,50]. Inhalation is the fastest and most effective way to induce responses in the central nervous system within a few seconds. The inhalation of the EO molecules delivers active volatile compounds to the brain and the circulatory system via (a) the olfactory lobe and (b) the respiratory system, respectively. The olfactory system begins with the nasal cavity which leads to the olfactory lobe located close to the brain. The olfactory lobe is connected to several brain areas, e.g., the hypothalamus and hippocampus. The volatile molecules in the citrus EOs enter through the nasal cavity, pass through the olfactory lobe, activate the sensory neurons present in the olfactory mucosa, and the axons of the sensory neuron cells ultimately deliver the signals to the central nervous system (CNS) [51,52,53]. The ‘activation’ is the initiation of electrical signals (by the odorant molecules) in the olfactory lobe. The signal is transmitted from the olfactory lobe to the olfactory cortex. The stimuli modulate specific physiological responses involving mood and behavioral actions (emotion and cognition), hormone production, regulation of body temperature, digestive reactions, memory, stress responses, sedation, sex stimulation, blood pressure, heart rate, etc. [54,55]. It has been observed that if the sense of smell is lost in patients with anxiety and depression, inhaled volatile molecules enter the lungs through the circulatory system via gas exchange and trigger the healing process. Another pathway of the EOs post inhalation is through the respiratory system involving gaseous exchange via diffusion into the blood circulation in the alveoli [49]. The EOs action toward brain functioning has been explained to take place via three basic mechanisms: (a) Activation of nasal olfactory chemoreceptors, (b) direct absorption of the EO active molecules into the neuronal pathway, (c) absorption of EO active molecules in the alveolar blood circulation. 

**Figure 2:**
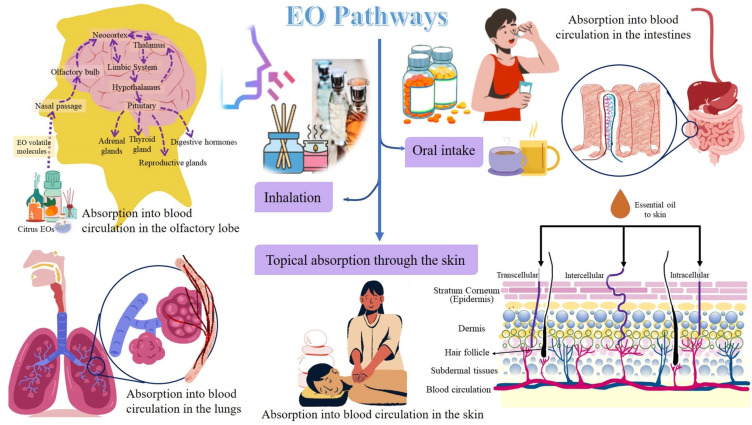
Pathways followed by citrus EOs for aromatherapy.

The pathways followed by citrus EOs are illustrated in Figure 2.

(a) Activation of nasal olfactory chemoreceptors

This involves the activation of nasal olfactory chemoreceptors and the consequent effects of the olfactory signals on the respective brain segments. The EO, upon inhalation, travels through the interiors of the nasal passage where the endothelium in the inner lining is thin and located close to the brain. Therefore, the EO molecules readily enter the local circulation and the brain. A particular odorant can activate a single or a set of OSN receptors and generate an electrophysiological signal for transmission into the brain. This is how different odors can be identified and differentiated. The olfactory epithelial layer is facilitated by different types of OSNs. A smell is identified by the activation of nasal olfactory chemoreceptors. The odorant molecules approach the olfactory epithelium and bind with the dendrite receptors present in the OSNs. This generates an electrophysiological signal via induction of an action potential. The axons of the OSNs extend and converge into the corresponding glomerulus cell. The latter is associated with a specific mitral and tufted cell. The signals are transmitted via dendrites of the glomerulus through connected mitral and tufted cells and eventually to the pyramidal neurons present in the olfactory cortex. In the cortex region, the transmitted electrophysical signals further stimulate the target regions in the brain [56,57]. The olfactory cortex of the brain is divided into other smaller regions, namely the piriform cortex, olfactory tubercle, and entorhinal cortex. Each of these regions project information to the amygdala (regulates aggression, eating, drinking, sexual behavior), hippocampus (regulates emotion, learning, memory, odor memory), and hypothalamus (regulates blood glucose levels, salt, blood pressure, and hormones) or ‘limbic system’. The olfactory signals directly transmits into the cortex and responses to the stimuli are expressed in terms of odor, memory, emotions, and endocrine functions [58].

(b) Direct absorption of the EO active molecules into the neuronal pathway

The small and volatile molecules present in the EOs can be transported either by extracellular or by intracellular transport mechanisms. In the intracellular mechanism, the EO active molecules directly pass through the neuronal pathway in the olfactory lobe and transmitted to the brain. The molecules bind with the olfactory receptor surface of the neurons and initiate receptor-mediated endocytosis (cells take in substances present outside the cell body by engulfing them in a vesicle which reopens inside the cell and the substance becomes a part of the cytoplasm). The molecules absorbed in the OSN are transported to the olfactory bulb along the axons by endosomes. In the extracellular transport mechanism, the EO active molecules pass through the paracellular cleft between the OSN and supporting cells and enter the lamina propria (connective tissues) through movement in the fluid. From lamina propria, the EO active molecules are further transported to perineural space along the axons and eventually arrive at the brain parenchyma. Finally, the EO active molecules enter across the blood–brain barrier and blood–cerebrospinal fluid barrier to spread into different regions in the brain. The EO active molecules now interact with the neurotransmitter receptors, namely transient receptor potential (TRP) channel proteins, glutamate, and γ-amino-butyric acid (GABA), 5-hydroxytryptamine (5-HT), and dopamine (DA), and produce anxiolytic and antidepressant effects [58].

(c) Absorption of EO active molecules in the alveolar blood circulation

**Figure 3:**
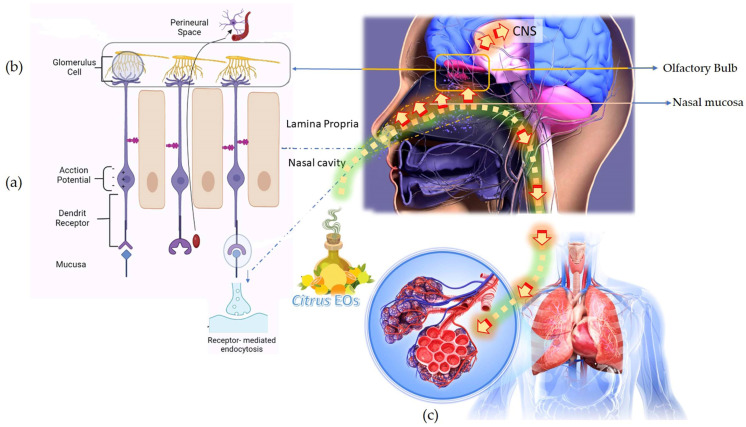
Inhalation of the citrus EOs and response delivery to the central nervous system through the olfactory lobe and respiratory and circulatory system. (**a**) Activation of nasal olfactory chemoreceptors (**b**) Direct absorption of the EO active molecules into the neuronal pathway (**c**) Absorption of EO active molecules in the alveolar blood circulation.

The EO vapor molecules, upon inhalation, travel to the lungs and induce an immediate and easing impact on breathing difficulties that appear during cold and congestion. The EO active molecules present in the inhaled vapor pass through the respiratory tract, enter the lungs, and reach the alveolar sacs where gaseous exchange between the cells of the alveoli and blood cells in the capillaries take place. Simultaneously, some molecules are also absorbed by the inner mucous linings of the respiratory tract, bronchi, and bronchioles. Deep breathing tends to increase the quantity of any EO components absorbed into the body by this route. EO active molecules enter the neuronal pathway, and absorption of the EO active molecules in the alveolar blood circulation is illustrated in Figure 3.

The soluble molecules present in the EO vapor carried with the inhaled air can cross the air–blood barrier. A majority of the EO components are lipophilic and hydrophobic in nature (lipid soluble terpene family). Lipophilic EO components can cross the blood–brain barrier and transport to the CNS [58] The EO action in aromatherapy through the inhalation process towards the brain functioning has been explained to take place via three basic mechanisms, viz., activation of nasal olfactory chemoreceptors and direct absorption of the active molecules. Aromatherapy is known to improve mood and certain mild symptoms of stress-related disorders, such as anxiety, depression, loss of appetite, loss of concentration, and chronic pain. The benefits of aromatherapy have been established by both the physiological and psychological effects upon inhalation of volatile EO components. The EO active components act via the limbic system, namely the hippocampal, the hypothalamus, and the pyriform cortex.

#### 4.2.2. Oral Intake

Citrus and its juice have been a major medicinal recipe for abdominal problems since ancient times in tropical and subtropical countries besides its use in foods, bakeries, and confectionaries. The lime fruits have been used for making anti-odorant agents due to the fragrance and freshness effects of their aromatic volatiles. Bergamot essential oils are utilized in pharmaceutical industries to absorb unpleasant odors of medicinal products and add antiseptic and antibacterial properties. In addition, the juice is added to drinking water, alcoholic, and non-alcoholic beverages to enhance flavor and antioxidants. The characteristic flavor of citrus oils is mainly due to the presence of certain components, namely linalool, citral, and linalyl acetate [59]. However, limonene and pinene present in the EO composition have not been much favored. Moreover, they are relatively unstable compounds and decompose when exposed to heat and light and they are removed from the oil to enhance the life of the products [59,60]. The roots of the lime tree have been used as a febrifuge and antipyretic in traditional medicine. The bark of the lemon tree is often boiled in water to obtain a decoction and taken as a remedy for gonorrhea and related disorders. In many tribal populations, the roots of the plant are dried and chewed for headache and vermifuge effects in the stomach and the intestines. The citrus EO components have several benefits when taken orally due to their antiviral, antiseptic, antimicrobial, astringent, restorative, stimulant, and antioxidant properties [12,46,48,61,62,63,64,65]. 

Bergamot EO possesses a bitter aromatic taste and a characteristic pleasant aroma. It is a popular pharmacopoeias in many countries. It has been also reported for its hypolipemic and hypoglycemics activities, anti-inflammatory, and anti-cancer properties [66,67,68,69,70]. In folk medicine in many countries, it has been popularly used for fever and parasitic diseases. Due to its significant antimicrobial properties, it has been found useful in treating infections in the mouth, skin, respiratory and urinary tract, gonococcal infections, leucorrhea, vaginal pruritus, tonsillitis, and sore throats [71]. BEO and vapors have been observed to exhibit resistance against common food-borne pathogens. The EO component linalool is reported to be the most effective antibacterial component [72]. BEO has also been reported for its antibacterial and anti-fungal activities against several microbial strains, such as *Campylobacter jejuni*, *Escherichia coli* O157, *Listeriamono cytogenes*, *Bacillus cereus*, *Staphylococcus aureus,* dermatophytes, and *Candida species*-induced infections [73,74,75]. BEO-incorporated chitosan-based films with concentrations, *viz*., 0.5, 1, 2, and 3% *w/w* have been reported to exhibit a significant dose-dependent inhibitory effect against *Penicillium italicum* [76].

Bergamottine (5-geranoxypsoralen), an important component in the Eos, is a natural furanocoumarin. It can be extracted from the pulp of pomelos and grapefruits and the peel and pulp of bergamot oranges. It has been found to decrease the electrocardiographic changes significantly during experimental studies in guinea pigs. The latter is typical of coronary arterial spasms and cardiac arrhythmias provoked by pitressin. Bergamottine is also found to increase the dose of ouabain required to induce ventricular premature beats, ventricular tachyarrhythmias, and death. The experimental studied suggest that bergamottine possesses potential anti-anginal and antiarrhythmic properties [77]. In another experimental model of rat angioplasty, a pretreatment with a volatile fraction of bergamot EO in a dose-dependent manner has been observed to reduce the neointima proliferation, together with the free radical formation and LOX-1 expression. Lectin-like oxy LDL receptor-1(LOX-1) is known to be involved in smooth muscle cell proliferation and neo-intima formation occurring in injured blood vessels [66]. Furthermore, the bergamot EO has also been observed to induce vasorelaxation of the mouse aorta by activating K^+^ channels and inhibiting Ca^2+^ influx [78]. The latter differentially modulates intracellular Ca^2+^ levels in vascular endothelial and smooth muscle cells [79]. These research findings altogether indicate that bergamot EO possesses potential activity as a vasodilator agent in cardiovascular diseases. Citrus EOs in oral administration has been observed to be beneficial in treating anxiety [80]. 

The citrus EOs undergo significant biotransformation after being absorbed in the digestive system which has been observed to alter their effects on health. When ingested orally, the EOs enter the digestive system and its components begin a wide range of actions. Primarily the monoterpenoids, namely *d*-limonene, carvone, *cis*- and *trans*- carveol (CAR), perillyl alcohol (POH), and geraniol have been observed to alleviate the carcinogenesis of exogenous substances. Other EO components, such as linalool and citral along with carvone and geraniol have been found to impart antimicrobial activities in the digestive system. The antimicrobial properties of the citrus EOs are attributed to the presence of abundant amounts of limonene and flavonoids in their composition [81]. Liver CYPs (Cytochrome P450) transform limonene into a variety of products. The CYPs act on various types of substrates or target molecules, and it has been observed that more than one P450 can act on the same type of substrate which produces multiple products from the same substrate. In human beings, the biotransformation of limonene occurs via four pathways, namely oxidation of endo- and exocyclic double bonds, oxidation of methyl side chain, and allylic oxidation of C6-ring [82]. The oxidation of the exocyclic double bond present in the limonene molecule produces Limonene (LMN)-8,9-OH, whereas the other three pathways produce perillyl alcohol (POH), perillic acid (PA), and *cis*- and *trans*- carveol (CAR). 

The biotransformation of α-pinene, the second major component of citrus EOs produces myrtenol, *cis*- and *trans*- verbenol. In addition, carene is transformed into caren-10-ol, caren-10-carboxylic acid and caren-3,4-diol [82]. Biotransformation of the citrus EOs alters its bioavailability. For example, the major component in the citrus Eos, limonene, is readily absorbed into the blood from the digestive tract. It is reported that the d-limonene (labeled with radioactive substance) is absorbed in the liver in 1.0 h with a peak concentration of 45.1 dpm (disintegration per min)/mg. Within the next 1.0 h, the peak concentration of the labeled d-limonene in adrenal glands and kidney was found to be 77.3 and 21.8 dpm/mg, respectively [83]. The biotransformation of limonene is a rapid process and the concentration of limonene, and its metabolites become undetectable within 24 h of ingestion (oral intake). The products of biotransformation of limonene (in citrus EOs) are excreted from the body via urine (~60%), feces, and breath after oral consumption [83]. 

**Figure 4:**
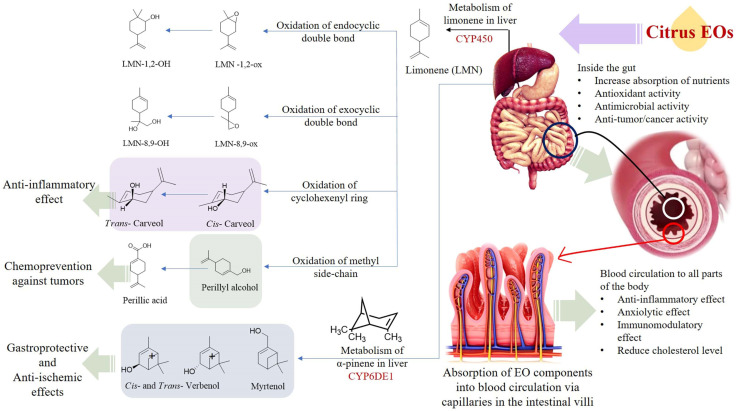
Metabolism of *d*-limonene and *α*-pinene in the liver, absorption of citrus EO components into the circulatory system through the intestinal villi.

**Figure 5:**
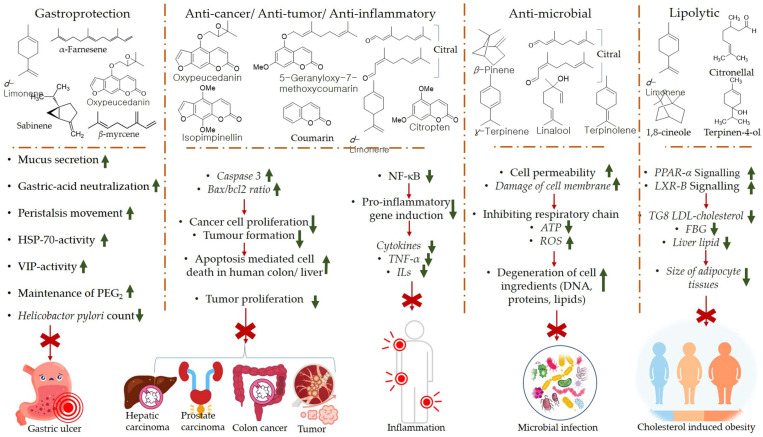
Mechanisms of gastroprotection, anti-cancer, anti-tumor, anti-inflammation, anti-microbial, and lipolytic actions of citrus EO components. Caspase (key apoptosis-inducing protein) [85,86,87,88,89,90,91,92]. Abbreviations; PPAR-α (Peroxisome proliferator-activated receptor alpha), bcl 2 (B-cell lymphoma protein 2), Bax (bcl 2-associated X), NF-ΚB (Nuclear factor-κB), LXR-β (Liver X receptor beta), TG8 LDL (Triglycerides 8 Low-density lipoprotein), FBG (fasting blood glucose), ROS (Reactive Oxygen Species), TNF-α (tumor necrosis factor alpha), ILs (Interleukins), ATP (Adenosine triphosphate).

The products of the citrus EOs post biotransformation exhibit certain health-promoting effects. Perillyl alcohol has been observed to reduce the incidence and diversity of colonic invasive adenocarcinoma in rats (induced by injecting methoxymethane (or azoxymethane (AOM) carcinogen). Furthermore, perillyl alcohol has been found to be more effective compared with limonene in terms of chemoprotection against malignant cancer [84]. The metabolism of *d*-limonene and *α*-pinene in the liver, and absorption of citrus EO components into the circulatory system through the intestinal villi is shown in Figure 4. The mechanisms of gastroprotection, anti-cancer, anti-tumor, anti-inflammation, anti-microbial, and lipolytic actions of citrus EO components are summarized in Figure 5.

The mechanism of chemoprotection by monoterpenes has been explained via several hypotheses, viz., G1block, induction of cell apoptosis or cell death, aggravation of stressed condition inside endoplasmic reticulum, and alteration in mevalonate metabolism pathway. Perillyl alcohol is believed to mainly block the modification of Ras oncoproteins; inhibit farnesyl-protein transferase (FPTase) and geranylgeranyl protein transferases (GGPTases), whereas other metabolites of limonene biotransformation, viz., cis- and trans-carveol (CAR) induce anti-inflammatory activity by suppressing the generation of superoxide dismutase (SOD) and nitric oxide and NF-κB signaling pathway. Furthermore, myrtenol and *cis*- and *trans*-verbenol (products of α-pinene biotransformation) have been observed to induce gastroprotective and anti-ischemic effects [82,93]. 

#### 4.2.3. Applications on Skin

Skin is the largest organ of the human body. Cell cytoplasm contains 90% of its composition as water, and therefore the skin acts as a protective barrier to resist water loss. However, the skin is semi-permeable to water and water-soluble substances. The barrier protection is attributed to the stratum corneum (the epidermis). It is an outer tough, durable keratinized layer with a thickness up to 20 layers of dead cells, and self-repairing. Beneath the epidermis is the dermis, a complex structure comprising lymph, blood vessels and capillaries, nerves, sweat and oil glands, hair follicles, collagen, elastin, fibroblasts, mast cells, and so on. Due to the lipids present in all cell membranes, the penetration of molecules through the dermis is relatively easier. The fundamental physicochemical properties of the external molecules which decide the rate and quantity of external molecules to penetrate the skin are the molecular weight of the molecule, its spatial structure and arrangement of functional groups, polarity, optical activity, liposolubility, coefficients of diffusion, dissociation, and so on. Due to the presence of lipids in the stratum corneum, the liposoluble compounds in the EOs make their way into the inner layers of the skin and finally reach into the blood stream.

Once the EO components penetrate the epidermis and enter the dermis, they are absorbed into the blood circulation and carried to every cell in the body. The hydrophilic and lipophilic molecules present in the citrus Eos can penetrate the skin through sweat gland openings, hair follicles, and sebaceous glands, respectively. The EO molecules progress through the passage between cells, i.e., fatty cement of the skin layers as well as through the cells themselves by intervening through the cell’s membrane made of phospholipids. The skin epidermis thickness is uneven in different parts of the body. For example, the skin epidermis of the forehead and scalp is relatively thinner and contains a large number of oil glands. Therefore, lipophilic molecules penetrate readily through the partial barrier and enter the blood stream. 

The lipophilic EO components are lipid soluble and tend to accumulate in lipid-rich areas of the body to form reservoir(s) and possibly to be sequestrated [94,95]. The EO constituent molecule reservoirs present in the outer layers of the epidermis and subcutaneous fat are retained in the fat for some time and do no disperse to the adjacent layers of the skin because of poor blood supply in this region [94]. Furthermore, the enzymes present in the skin participate in regulating (activation/inactivation) the natural chemicals present in the body, such as hormones, steroids, and inflammatory mediators as well as externally applied chemicals, such as medicines/drugs and EO components. In addition, the enzymes also participate in the metabolism of EO components which may result in the change of molecular structure of the original compound. The latter changes the effect on the body. The skin enzymatic activities vary differently in different age group individuals which define skin elasticity, dehydration, damage, broken, pigmentation, inflammation, diseases, and so on [94].

Bergamot EOs (BEO) have been a part of homemade ointments, soaps, toiletries, bodywash, shampoos, anti-dandruff and hair care products, masks and cleansers, candles, and massage oils (a mixture of oils) employed for skin disinfection [96], as an astringent [97], antiseptic or aid for healing minor wounds [98], insect bites, sunburn, aromatherapy massage, and cosmetics [99]. BEO aromatherapy massage as a complementary therapy to the patients suffering from cancer has been observed to induce relaxation from symptoms of clinical anxiety and depression for up to two weeks [100]. Furthermore, aromatherapy involving BEO has been observed to help in improving mood, and symptoms of mild stress and facilitating sleep induction [101]. The commonly used EOs from citrus in aromatherapy (in the form of aroma-sticks) in clinical studies are lemon (*Citrus limon* (L.) *Osbeck*) [102], bergamot (*Citrus bergamia*) [103] and orange sweet (*Citrus sinensis* (L.) *Osbeck*) [104] along with EOs from other herbs, such as lavender (*Lavandula angustifolia Mill*.), frankincense (*Boswellia carterii*), and peppermint (*Mentha piperita*) [105,106]. 

BEO is reported to exhibit anti-inflammatory activity while conducting a carrageenan-induced rat paw edema test. The optimal response for the anti-inflammatory activity was observed with a 0.10 mL/kg dosage injected intraperitoneally while the median effective dose was 0.079 mL/kg [73]. The absorbed EO and its components penetrated the skin and can be detected in exhaled air of the breath within 20–60 min. For example, the times taken to detect citrus EO components in exhaled breath post penetration into the skin are 1,8-cineole and α-pinene (20 min); linalyl acetate, geranyl acetate (between 20 and 40 min), bergamot, and lemon oils (40 and 60 min, respectively), and geraniol and citral (up to 2 h) [94,107]. 

Lemon EO has antioxidant properties, i.e., fighting free radicals which cause premature aging of the skin, and is therefore a popular ingredient in skincare products. The antibacterial property of lemon EOs is attributed to its components, viz., citric acid, limonene, and pinene. This makes it a suitable component in formulating cleansers, body washes, and soaps, as it helps in removing bacteria, and other microbes from pores of the skin of acne-prone oily skin. Furthermore, lemon EOs also possess astringent properties which are effective in closing the pores in the skin and preventing the blockages from being inflamed. For a typical formulation to be utilized in topical applications, citrus EOs in a blend with other EOs, such as lavender and chamomile EOs, are employed for calming skin inflammation and reducing redness. In skin lotions and ointments, the citrus EOs are mixed with a carrier oil, such as jojoba oil or olive oil to dilute the potency of the oil for applications at sensitive areas such as the face, neck, and chest. Some citrus EOs, e.g., EOs from bergamot, lemon, and grapefruit exhibit phototoxic effects (e.g., skin-irritation, damage) upon exposure to sunlight/UV rays in the Sun’s radiation owing to furanocoumarins, especially 5-MOP (5-methoxypsoralen or bergapten) present in the EO composition. Removal of psoralen (the parent compound in a family of naturally occurring linear furanocoumarins) from the citrus EOs-containing formulations has been found to eliminate the possibility of phototoxicity [108].

**Figure 6:**
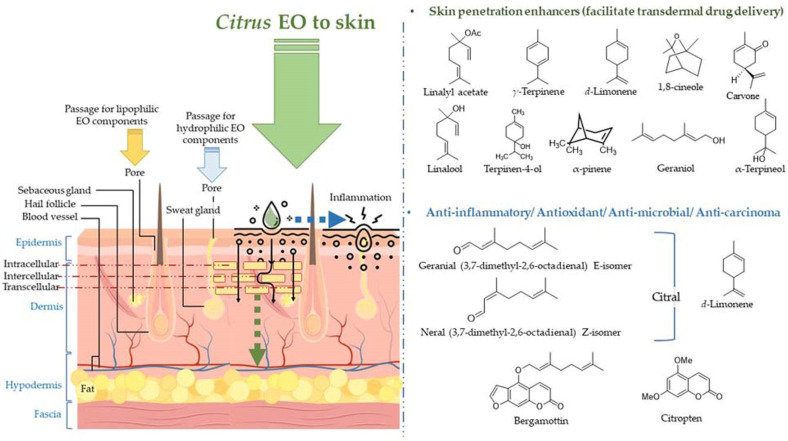
Absorption of citrus EOs to the deeper layers of the skin, molecular structures of the skin penetration enhancers (frequently employed in topical lotions/ointments for facilitating transdermal drug delivery), and molecules participating in anti-inflammatory, anti-microbial, and anti-carcinoma activities.

The volatile constituents in the EOs penetrate the skin through deeper layers of the skin via different mechanisms of action, viz., interaction with the highly ordered intercellular lipid structure in stratum corneum (SC), and interaction with intercellular proteins resulting in conformational changes, and the latter increases permeability of the skin [109]. The penetration of the EO components also forms a pathway for different drugs (hydrophobic and hydrophilic), and vitamins in the topical formulation to enter lower skin layers [109,110,111,112]. Furthermore, the EO components are rapidly metabolized, not accumulated in the skin and the body, and rapidly excreted after application to the skin; therefore, regarded as safe penetration enhancers [109]. The absorption of citrus EOs to the deeper layers of the skin, molecular structures of the skin penetration enhancers (frequently employed in topical lotions/ointments for facilitating transdermal drug delivery), and molecules participating in anti-inflammatory, anti-microbial, and anti-carcinoma activities are displayed in Figure 6.

Terpenes in citrus EOs are proven as promising nontoxic, non-irritating penetration enhancers for both hydrophilic and lipophilic drugs [113,114]. Some of the well-known penetration enhancer molecules present in the citrus EOs are *d*-limonene, α-pinene, α-terpineol, carvone, and 1,8-cineole. The terpenes exhibit a significantly efficient fluidizing effect on the lipid bilayer structure. Limonene has been found to induce change in the barrier structure of the skin in the presence of ethanol and facilitate the permeation of EO components and drug molecules utilizing its affinity with alcohol. In addition, sesquiterpenes also have been found to increase the penetrability of the skin by altering the structure of intercellular lipid bilayer and formation of a complex with the drug molecule [109,111].

## 5. Aromatherapy Using Citrus EOs for Health and Treatment of Diseases

### 5.1. Oxidative Stress

Free radicals, such as reactive oxygen species (ROS), and reactive nitrogen species (RNS) are produced during cellular aerobic respiration in mitochondria (endogenous). ROS are also produced when skin is exposed to ultraviolet (UV) light (UV-A; 320–400 nm, and UV-B; 290–320 nm) and this is known as the exogenous origin of free radicals. In addition to the ROS, superoxide anion radical (*O_2_^•–^), hydrogen peroxide (H_2_O_2_), hydroxyl radical (*OH), singlet oxygen (*O_2_), lipid peroxides (LOOH), and their radicals (LOO*) are also formed which participate in the process of skin aging, phototoxicity, induction of inflammation, and inflammation-induced malignant tumors [115,116,117,118,119]. The free radicals attack and degenerate structural molecules, such as collagen; and functional biomolecules, such as RNA and DNA, fatty acids, proteins, and other essential molecules. This gives rise to several complications which result in aging, inflammation, cancer, Alzheimer’s disease, Parkinson’s disease, diabetes, atherosclerosis, liver disease, etc. Oxidative stress is one of the main reasons behind allergic and inflammatory skin diseases, e.g., atopic dermatitis, urticaria, and psoriasis. Furthermore, microbial infections, e.g., that are caused by *S. aureus*, may worsen the damaged and lesioned skin by the production of ROS [120].

Aerobic respiration at the cellular level takes place in the mitochondria. The latter is a double-walled organelle (in eukaryotic cells) which carries out aerobic respiration and produces adenosine triphosphate (ATP). ATP is the utilizable form of the chemical energy consumed by the cell in its various functions. In diseased conditions, such as Alzheimer’s disease, dementia, or aging, the mitochondria undergo a dysfunctional stage during which oxidizing free radicals are generated in excessive amounts which eventually leads to oxidative stress and oxidative damages to essential molecules in the cell and ultimately pathological abnormalities. Beta-amyloid (Aβ) is an initiator of reactive oxygen species (ROS) and reactive nitrogen species (RNS). The free radicals attack and damage the essential molecules present in the cell including membrane lipids, and cellular organelles and generate mitochondrial toxins, such as hydroxynonenal (HNE) and malondialdehyde. When the membrane bound ion selective ATPase is damaged because of oxidative stress, it stimulates the NMDA receptors, membrane attack complex (MAC), and ion-specific Aβ pore formation. As a result, an influx of calcium ions increases and consequently cytosolic and mitochondrial calcium load. In the next stage, cellular amyloid targets essential enzymes, namely cytochrome-C oxidase, α-ketoglutarate dehydrogenase, pyruvate dehydrogenase, and manganese superoxide dismutase (MnSOD). This causes damage to the mitochondrial DNA and ultimately fragmentation of the structure. Aβ stimulates stress-induced protein kinases-p38, c-jun N-terminal kinase (JNK), and tumor suppressor protein (P^53^) leading to apoptosis or cellular damage. 

In natural and healthy physiological conditions, the free radicals generated are neutralized to non-radical forms under the action of certain enzymes, e.g., catalase (CAT) and hydroxy peroxidase. In acute and chronic cases or low immunity, the production of free radicals becomes radically high. To elaborate on this, products of lipid peroxidation stimulate phosphorylation and aggregation of tau proteins. The latter inhibits complex-I in a cell under oxidative stress, and excessive quantities of ROS and RNS are produced at complexes I and III. In the final stage, mitochondrial membrane potential (MMP) drops, and the permeability-transition pores (ψm) opens. The latter results in the activation of caspases and cellular damage. Ultimately, the reactive species (ROS and RNS) readily initiate oxidative degradation of somatic and brain cells (neural, microglial, and cerebrovascular cells). In such conditions, supplementary administration of free radical scavengers is recommended [58,121]. 

The citrus EOs possess antioxidative properties due to the ability of the component molecules to donate a hydrogen atom, or an electron to the free radicals which can delocalize the unpaired electrons (in conjugated/aromatic structure), thus neutralizing the free radicals and protecting the biological molecules from being damaged by oxidation or oxidative stress. The EO components also interfere with lipid metabolism in animal tissues by upregulating the activities of antioxidative enzymes, such as superoxide dismutase, catalase, and glutathione peroxidase. This results in the inhibition of the formation of reactive oxygen species and oxidation of polyunsaturated fatty acids which give rise to off-flavors in the food materials [122,123]. Inhalation of Citrus EOs can increase the amount of GSH and cause a reduction in lipid peroxidation in the brain, and it helps prevent DNA cleavage and cell apoptosis by scavenging free radicals (ROS) via antioxidant effects. Inhalation of EOs augments the level of antioxidant enzymes involved in the immune system, e.g., superoxide dismutase (SOD), glutathione peroxidase, and catalase (CAT). It has been found that terpenes present in the citrus EOs can reduce inflammation symptoms by decreasing/inhibiting the release of pro-inflammatory cytokines, such as NF-κB (nuclear transcription factor-kappa B), IL-1β (interleukin-1β), and TNF-α (tumor necrosis factor-alpha) [124]. 

In addition to monoterpene hydrocarbons, limonene can also inhibit the production of pro-inflammatory cytokines in lipopolysaccharide (LPS)-induced inflammation symptoms, and the production of ROS in H_2_O_2_-induced oxidative stress and wound healing. EOs obtained from bergamot and sweet orange have been found to heal acne vulgaris caused by excessive secretion of androgen by reducing the growth rate of as well as secretion from, sebaceous glands. This activates the inhibition of triglyceride (TG) accumulation and the release of inflammatory cytokines in the sebaceous glands. This results in apoptosis in sebaceous glands leading to a decrease of T/E_2_ ratio. The EOs act to lower the IL-1α levels in sebaceous glands which help in improving acne lesions by alleviating inflammatory responses [121,125,126]. Another study investigating limonene’s anti-inflammatory response on human eosinophilic leukemia HL-60 clone 15 cells revealed interesting results. Hirota et al. [127] reported that a low concentration of limonene (7.34 mmol/L) can inhibit ROS production for eotaxin-stimulated HL-60 clone 15 cells. 

**Figure 7:**
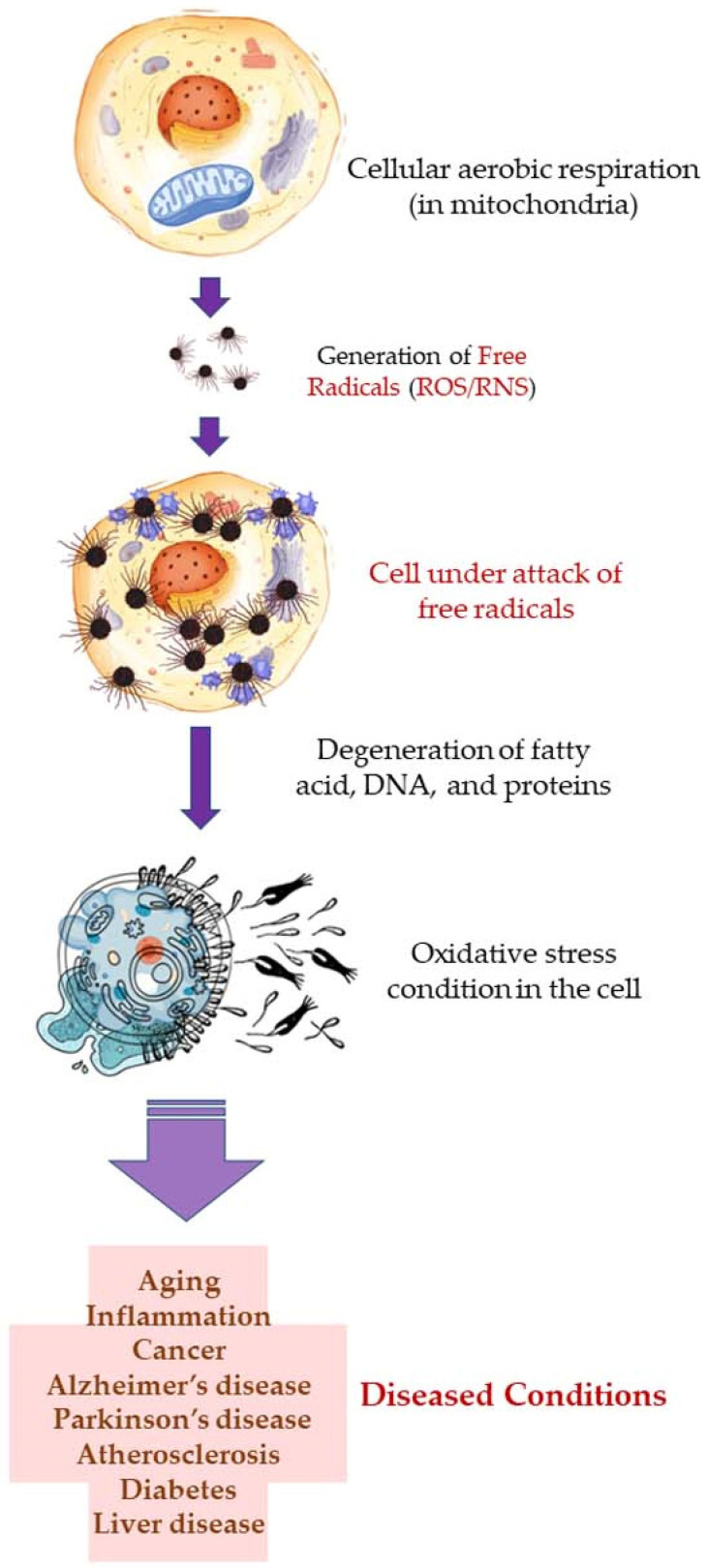
Oxidative stress in the cell: Events and consequences-I: Somatic cell.

**Figure 8:**
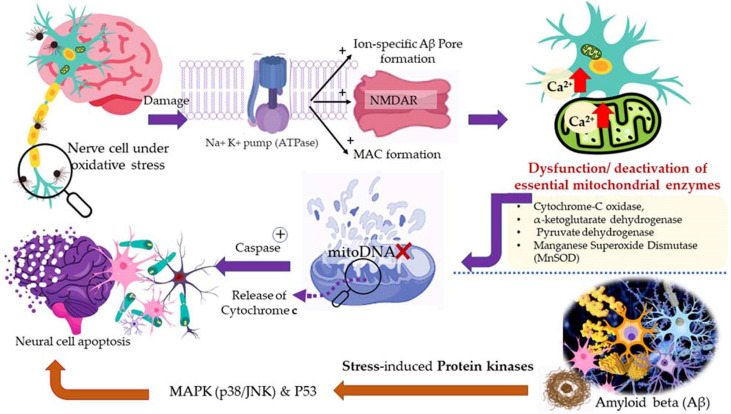
Oxidative stress in the cell: Events and consequences-II: Nerve cell.

**Figure 9:**
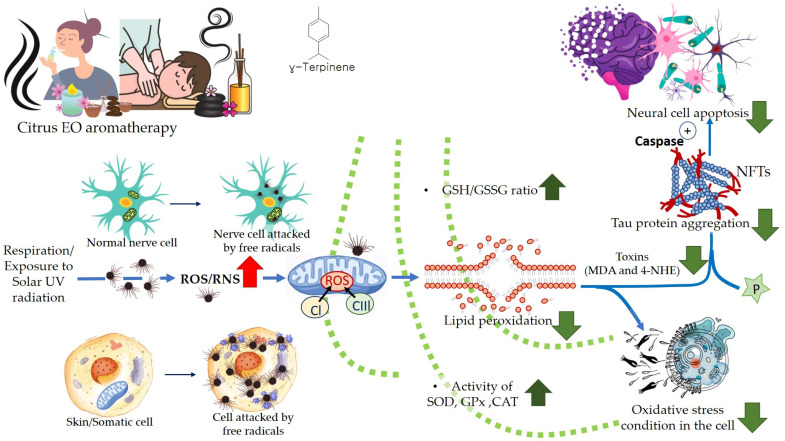
Therapeutic effect of citrus EOs aromatherapy.

A higher limonene concentration of 14.68 mmol/L was found to diminish diesel exhaust particles (DEP)-induced MCP-1 production significantly, indicating that the antioxidant activity of limonene can help restrict monocyte infiltration into the lungs and prevent migration of eosinophil, protecting asthmatic lungs and prevent damage from DEPs in the lung. Furthermore, NF-κB formation was also diminished upon the addition of proteasome inhibitor MG132. The limonene can inhibit DEP induced p38 MAPK signaling pathway and inhibit eotaxin-induced chemotaxis by eosinophils [127]. Citrus EO components exhibit antioxidative activities against the oxidation of linoleic acid. In addition, antioxidant activities have also been reported against in vitro oxidation of human low-density lipoprotein induced by Cu^2+^, and 2, 2′-azobis (2-aminopropane) hydrochloride [128]. The antioxidant properties of citrus EOs are attributed to the presence of phenolic compounds in their composition. Monoterpene hydrocarbons (limonene, thujene), and oxygenated monoterpenes (monoterpenes with different functional groups, such as phenols, alcohols, aldehydes, ethers, esters, and ketones) contribute significantly to the antioxidant properties of the citrus EOs [129]. The events and consequences of oxidative stress in a somatic and nerve cell, and the therapeutic effects of citrus EO aromatherapy are displayed in Figure 7, Figure 8 and Figure 9.

Thujene, a monoterpene, has been reported to exhibit good antioxidant activity due to its ability to quench singlet oxygen efficiently [130]. The alcohol compounds, e.g., carveol and perillyl alcohol; ketones, e.g., carvone and aldehydes, perillyl aldehyde; esters, e.g., citronellyl acetate, geranyl acetate, neryl acetate exhibit good antioxidant activities. Among the compounds, γ-terpinene, geranial, R-(+) limonene, and β-pinene have been reported for possessing the highest antioxidant capacities [131,132,133].

### 5.2. Stress-Related Disorders/Mood Disorders

Stress-related disorders or mood disorders have become very common in everyday life. Mood disorders include several psychiatric illnesses which significantly (sometimes severely) impact the mood related function of an individual (patient). The disorders are characterized by cognitive deficits such as impaired learning, loss of memory, and inability to focus/concentrate. Sudden, significant, and persistent changes in emotions or state of mind, sadness, anxiety, depression, sleep disorders, and insomnia are symptoms associated with chronic stress or trauma. Mood disorders originate from physiological, psychological disturbances, organic damage, nerve injury, side effects of medications, chronic stress, etc. Depression is characterized by a combination of symptoms associated with traumatic emotions (sadness and anhedonia), cognition deficit, and somatic symptoms (change in appetite, such as over/under eating), sleep disorders, insomnia, melancholy, hopeless, despair, detachment from daily life/routine activities, fatigue, and even suicidal tendencies. Anxiety is mainly caused by physiological and psychological disturbances, e.g., emotional, behavioral, environmental, somatic, and social elements. When any of these elements invoke unpleasant situation or sensations, fret, phobias, disquietedness, or restlessness, the human mind enters a stressed condition or anxiety. Prolonged stressed conditions lead to a stage when the person faces the onset of anxiety symptoms, such as unusual panic situations characterized by hypertension, sweating, palpitation, chest pain, migraine, papillary dilation, shortness of breath, and so on [134,135]. According to a WHO report, more than 260 million people are suffering from depression with varying levels and approximately 800,000 people die by committing suicide every year [136]. Furthermore, more than 50 million people are known to be suffering from dementia/Alzheimer’s disease which is projected to rise in number up to 82 to 152 million by the years 2030 and 2050, respectively. A stressed or diseased person finds it difficult to perform his/her daily life and respond approximately to the problems, challenges, or important events on time. Moreover, the disease further progresses with loss of memory. In the pathological aspect, the diseased person is diagnosed by the presence of amyloid plaques, neurofibrillary tangles, and loss of neural transmission in the brain [137,138]. Insomnia patients have common symptoms of depression and anxiety, and no single medication is known to cure this condition accurately. Insomnia is also characterized by acute sleep disorder. Prolonged disturbance in sleep patterns may result in high blood pressure, cardiovascular diseases, and severe risks of acute mental illnesses [139,140,141].

Bergamot oil has been found to reduce blood pressure and heart rate and help induce sleep and relief from restlessness. EOs extracted from sweet orange and lavender EO have been observed to improve sleep quality and provide relief from tiredness in hemodialysis patients [142]. Takeda et al. carried out a study on inhalation aromatherapy in elderly dementia patients by applying EO drop on towels covering their pillows during their sleep time. The researchers recorded a better sleep latency and improved total sleep time and effectiveness of sleep among the treated people [143]. The aromatic EO molecules enter limbic system in the brain via nasal passages and stimulate GABA receptors in the hypothalamus. The overall process induces and maintains restful sleep [144]. Citrus EO (with 95% citral in the composition) has been observed to induce a pleasant mood in people suffering from sadness [145]. The molecular pathways involved in the pathophysiology of depression include the hypothalamic–pituitary–adrenal axis, sympathetic nervous system, monoamine neurotransmission system (e.g., serotonergic (5-HT), dopaminergic (DA), and GABAergic pathways), cyclic adenosine monophosphate (c-AMP) response element-binding (CREB) protein signaling pathway [58,146,147,148,149,150,151,152]. According to neurotropic hypothesis, depression is associated with a deficit of neurotropic factors caused by prolonged exposure to stress which results in loss of neural plasticity [153]. Brain-derived neurotropic factors (BDNF), a protein in the brain produced by BDNF gene, and neurotrophins, a class of growth factors, promote the growth of the neurons and maintain adequate neural plasticity. During depression, the level of BDNF in the serum decreases. Therefore, deficiency of neurogenesis or production of new neurons in the brain hippocampus is a major reason behind depression. EO-based aromatherapy involving EOs of lavender, lemon, and bergamot has been reported to prevent negative symptoms of depression, such as deficiency of neurogenesis, suppressed dendritic growth of immature neurons, and low serum BDNF levels in the brain hippocampus [154,155,156,157]. In a clinical study involving patients diagnosed with stress- and depression-related symptoms, such as attention deficit and hyperactivity disorder, four weeks employing EO-based aromatherapy resulted in a decrease in the level of anxiety and depression and a simultaneous increase in blood plasma BDNF levels in the brain hippocampal tissues [157]. Moreover, regarding neurogenic and enhancing neurotropic factors in the human brain, citrus EOs have also been observed to participate in the regulation of the neuroendocrine system. Depression and anxiety disorder release the stress hormone cortisol. Aromatherapy involving lavender EO has been observed to downregulate the release of stress hormones and a decrease in salivary and serum cortisol levels was recorded [48,158]. In addition, bergamot EO and grapefruit seed EO have also been reported to induce lowering of cortisol levels in the blood, thereby lower stress related symptoms. There have also been recorded improved coronary flow velocity and enhancement in relaxation. Bergamot EOs have been observed to cause an alteration of HPA axis and attenuate the rise of corticosterone levels in the blood [159]. Lemon EOs have been recorded to produce antidepressant effects in terms of accelerated turnover of dopamine in the brain hippocampal region establishing therapeutic effects of EOs in healing the patients from depression and related symptoms [58].

Anshen EO, a mixture of EOs from lavender, sweet orange, and sandalwood, has been observed to have anxiolytic, antidepressant, sedative, and hypnotic effects. Researchers have performed sleep latency and sleep duration experiments, where they compared diazepam—generally used to treat insomnia—with anshen EOs [160]. Mouse brain responses were analyzed using ELISA test to detect changes in 5-HT and GABA levels. The results showed a significant decrease in impulsive activities and reduced sleep potential. An increase in the levels of 5-HT and GABA was observed in the mouse brain. Anxiolytic effects of BEO (1.0, 2.5, and 5.0% *w*/*w*) were studied by administering it to rats subjected to anxiety-related behaviors, the elevated plus-maze, and the hole-board tests, and then measuring the stress-induced levels of plasma corticosterone in comparison with the effects of diazepam. BEO (2.5%) and diazepam exhibited anxiolytic-like effects and attenuated the corticosterone response to acute stress [159]. After perfusion into the hippocampus via the dialysis probe (having volumetric flow rate 20 μL/min), BEO produced a dose-dependent and Ca^2+^-independent increase of extra cellular aspartate, glycine, taurine, GABA, and glutamate [161]. Inhalation of orange EO for 90 s has been observed to cause a significant decrease in oxyhemoglobin concentration in the right prefrontal cortex of the brain which increases comfortable, relaxed, and natural feelings [104]. Osbeck EO from *Citrus sinensis* Osbeck is found to exert antidepressant effects, being suitable to treat minor stress. The effects of Osbeck EO inhalation on CUMS (Chronic Unpredictable Mild Stress) mice were found to tackle depression along with decreased body weight, interest, movement, and dyslipidemia. Limonene is not metabolized in the brain immediately after inhalation. An in-depth study revealed that limonene is significantly effective as an antidepressant and shows healing progress in the neuroendocrine, neurotrophic, and monoaminergic systems [17]. 

Moradi et al. [162] conducted a study on patients who underwent coronary angiography. The patients were divided into two intervention groups, each comprising 40 patients. Patients of the test group inhaled EO from *Citrus aurantium* for 15–20 min about 60 min before the procedure. In the control group, distilled water was used instead of EO. Following *Citrus aurantium* EO inhalation, noticeable responses were observed. Vital signs of anxiety such as pulse rate, systolic blood pressure (SBP), and diastolic blood pressure (DBP) were significantly decreased after the intervention [162]. Li et al. [163] compared the effects of an essential oil mixture (EOM) (a mixture of *Citrus sinensis* L., *Mentha piperita* L., *Syzygium aromaticum* L. and *Rosmarinus officinalis* L.), with peppermint EO on physical exhaustion in two rat groups. After swimming, the two rat groups were maintained in an environment of EOM and peppermint EO, respectively. Various body parameters were studied after three continuous days of nebulization. Blood lactic acid (BLA) and malondialdehyde (MDA) levels were found to decrease in both groups. An improved duration of fatigue and increased superoxide dismutase (SOD) activity were observed in both groups. The results observed in the EOM group were noticeable, such as an increase in blood glucose and a reduction of blood urea nitrogen (BUN) and glutathione peroxidase (GSH-PX). This study determined that exercise-induced fatigue can be effectively relieved by inhalation of EOs [163]. Another study was performed on Swiss male mice to observe the neurotransmission contribution of nitric oxide when *C. sinensis* EO was used for its anxiolytic effects. To perform this study, mice were placed in an environment of *C. sinensis* for inhalation of EOs at different concentrations. Nitric oxide was used as a precursor to observe the mediation behavior of the nitrergic system, and it was found to play a significant role in the anxiolytic effect of *C. sinensis.* Bergamot essential oil (BEO), obtained from the fruit of *Citrus bergamia*, is used in aromatherapy as a pain reliever, improves sleep disorders, and reduces anxiety. BEO can induce neurotransmission which is associated with its anxiolytic-relaxant effects. Anxiolytic effects are shown to be the result of the collaborative action of BEO and the 5-hydroxytryptamine (5-HT) 1A along with the involvement of multiple and complex mechanisms [19].

### 5.3. Diseased Conditions

#### 5.3.1. Neurogenic Inflammation

Neurogenic inflammation is inflammation in neurons caused by the release of pro-inflammatory mediators, namely Substance P, calcitonin gene-related peptide (CGRP), neurokinin A (NKA), and endothelin-3 (ET-3). The release of pro-inflammatory mediators in the neurons is stimulated by the activation of ion channels (transient receptor potential ion channel-1 or TRPA-1) in response to harmful/unpleasant environmental stimuli. Acute neurogenic inflammation is caused by the activation of TRPA-1 channels induced by LPS. Following the release of inflammation causing neuropeptides is the release of histamine from the mast cells present in the vicinity of the affected neurons. The latter stimulates release of Substance P and calcitonin gene-related peptide, thereby establishing a bidirectional link between histamine and neuropeptide in the causation of neurogenic inflammation. Approximately 25% of migraine cases lead to temporary dysfunction of the central nervous system associated with visual field disturbances, sensitivity to light/sound, nausea, and/or vomiting [164]. 

**Figure 10:**
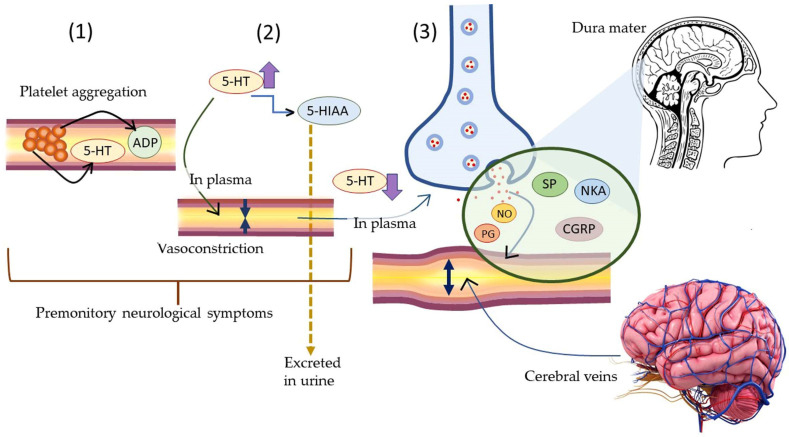
The pathophysiological mechanism of migraine induced by 5-HT. (1) Platelet aggregation trigger release of 5-HT and ADP in blood plasma. (2) High level of plasma 5-HT causes reversible vasoconstriction followed by conversion of 5-HT to its metabolite 5-HIAA. The latter is excreted in urine. (3) Low level of plasma 5-HT stimulates perivascular neurons to release neuropeptides (NO, PG, SP, NKA, CGRP) causing vasodilation of cerebral veins. The latter leads to migraine symptoms.

**Figure 11:**
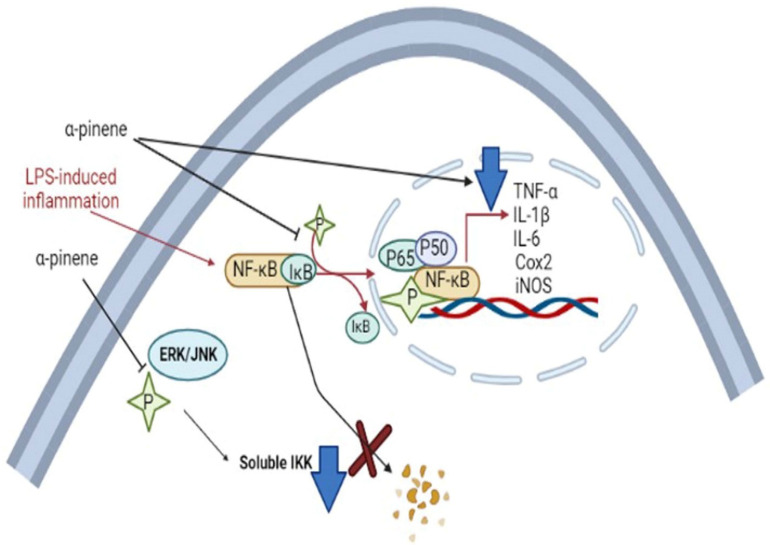
Neuroprotective mechanisms of *α*-pinene in migraine [168]. The α-pinene can reduce LPS-induced inflammation in macrophages. α-pinene can block phosphorylation of MAPKs (ERK/JNK) in macrophages and reduce the level of active (soluble IKK). This can prevent degradation of the NF-ĸB/IĸB complex. Also, α-pinene can hinder NF-ĸB phosphorylation and formation of the P65/p50/NF-ĸB complex that leads to its nuclear translocation and induction of inflammatory genes to generate cytokines. Abbreviations; TNF-α (tumor necrosis factor alpha), IL-1β (Interleukin-1β), IL-6 (Interleukin), Cox-2 (Cyclooxygenase-2), Inos (Inducible nitric oxide synthase).

Terpenes and terpene derivatives have been investigated for anti-inflammatory bioactivities. In this regard, limonene, *α*-pinene, *β*-caryophyllene, and *β*-myrcene have been most preferred for migraine cases [165]. Alpha-pinene (*α*-pinene) present in citrus EOs has been found to reduce NF-κB/p65 nucleus of LPS-stimulated THP-1 cells and increase the cytoplasmic concentration of Iκ-Bα protein. Alpha-pinene (*α*-pinene) also significantly decreases the levels of IL-6, TNF-α, and NO, as well as the expression of iNOS and Cox-2 induced by LPS. An in vitro study on *d*-limonene activity revealed an increase in IL-10/IL-2 ratio, consequently enhancing IL-10 levels. The latter is a cytokine synthesis inhibitory factor and inhibits proinflammatory Th1 cytokine production (IL-2) [166]. Furthermore, *d*-limonene epoxide has been observed to prevent the release of inflammatory mediators, inhibit vascular permeability, reduce migration of neutrophils, and display systematic and peripheral analgesic effects towards the brain’s opioid system (associated with regulating pain, reward, and addictive behavior) [167]. The pathophysiological mechanism of migraine induced by 5-HT and neuroprotective mechanisms of α-pinene in migraine are displayed in Figure 10 and Figure 11, respectively.

Neurogenic inflammation further causes conditions for the pathogenesis of several other neurogenic diseases, namely multiple sclerosis, migraine, psoriasis, asthma, vasomotor rhinitis, and so on. In migraine, the stimulation of the trigeminal nerve takes place which releases neuropeptides, such as Substance P, nitric oxide, 5-HT, vasoactive intestinal polypeptide, neurokinin A, and CGRP which eventually results in “sterile neurogenic inflammation”. The release of Substance P stimulates the production of several other pro-inflammatory cytokines, namely interleukins (IL-1, IL-6), and TNF-alpha (TNF-α). Migraine is characterized by a strong headache accompanied with nausea, vomiting, and sensitivity to light which may persist up to 72 h or longer. The phases in migraine can be explained to take place in four stages, *viz*., (a) prodrome: this stage persists for a few hours to few days and is characterized by irritability, depression, yawning, nausea, fatigue, muscle stiffness, difficulty in concentration and sleep; (b) aura: this persists for 5 to 60 min and is characterized by visual disturbances, temporary loss of sight, numbness in hands and feet, and tingling sensations in the body; (c) headache; this persists for 4 to 72 h and is characterized by throbbing pain, sensitivity to light, noise, odors, nausea, vomiting, giddiness, insomnia, neck and body pain and stiffness, and burning; and (d) postdrome: this is characterized by an inability to concentrate, fatigue, and lack of comprehension.

#### 5.3.2. Dementia, Alzheimer’s Disease (AD), and Parkinson’s Disease (PD)

Alzheimer’s disease is an age-related neurodegenerative disorder characterized by gradual memory loss and dementia. It also shows cognitive dysfunctions and turbulent behavioral patterns. At a physio-chemical level, it is diagnosed by scarcity in cholinergic neurotransmission in the cranial (brain) nerves, cognitive dysfunction, behavioral turbulence, gradual memory loss, accumulation of amyloid plaques (amyloid-β, Aβ) and neurofibrillary tangles (NFTs) in the specific brain areas, reduced glutathione (GSH) content in the hippocampus, mitochondrial dysfunction in the cells, and excess production of free radicals leading to oxidative stress [169]. The cholinesterase (ChEs) enzyme hydrolyses acetylcholine (Ach) into choline and acetate and the concentration of Ach neurotransmitter molecules in the brain drops resulting in the termination of neurotransmission. Acetylcholine is involved in the key function of learning and memory. In addition, monoamines, *viz*., dopamine and serotonin (5HT), released in the brain are also attributed to learning and memory. A decrease in the dopamine amount in the brain, and consequently, functional degradation of dopamine receptors has been identified as one of the common causes of Parkinson’s disease and Alzheimer’s disease [170]. For symptomatic management of AD, inhibitors of acetylcholinesterase (AChE) and butyrylcholinesterase (BChE) enzymes responsible for the degradation of the essential neurotransmitter acetylcholine (ACh) are considered for the development of anti-AD drugs. The choline esterase inhibitors reversibly bind to the active sites of acetylcholinesterase (AChE)/butyrylcholinesterase (BChE) enzymes. As a result, the hydrolytic degradation of ACh neurotransmitter molecules into choline and acetate is inhibited. Consequently, the concentration of ACh increases at the synaptic gaps in cholinergic neurons in the hippocampus cerebral cortex and some parts of the new striatum. Other neurodegenerative pathological conditions in patients suffering from AD include an increase in monoamine oxidase (MAO) activity and lipid oxidation induced by Fe^2+^ ions. The increase of MAO deactivates neuroactive amines, such as serotonin, dopamine, and norepinephrine, and enhances the production of free radicals (or ROS) in the patient’s brain [171]. Fe^2+^ ions have the ability to cross the blood–brain barrier which induces lipid oxidation via Fenton’s reaction. This leads to an abundance of polyunsaturated fatty acids in the brain tissues and causes vulnerability to free radical attacks. The latter causes the formation of radical species, e.g., MDA which participates in neurodegeneration. As a remedy, if an antioxidant mechanism stops or inhibits the lipid peroxidation products (MDA), it is possible to deplete the concentration of free Fe^2+^ ions in the cytosol. Consequently, the level of oxidative stress decreases in the brain as well as in the entire body [172,173,174,175,176,177]. 

**Figure 12:**
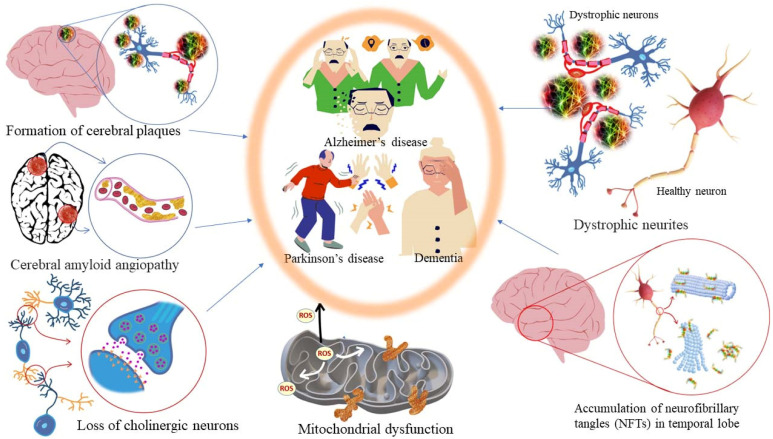
Pathological targets in diseased conditions of dementia, Alzheimer’s, and Parkinson’s.

**Figure 13:**
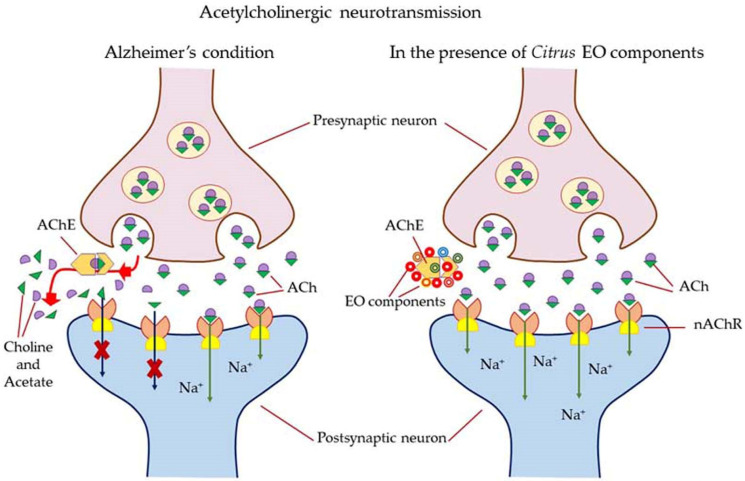
Mechanism of action of citrus EOs to inhibit acetylcholinesterase (AChE), thereby increasing levels and duration of acetylcholine in the brain and assisting with cognition (learning and memory retention). Abbreviation; ACh—acetylcholine; AChE—acetylcholinesterase; nAChr—nicotinic acetylcholine receptors; EOs—Citrus essential oil components.

**Figure 14:**
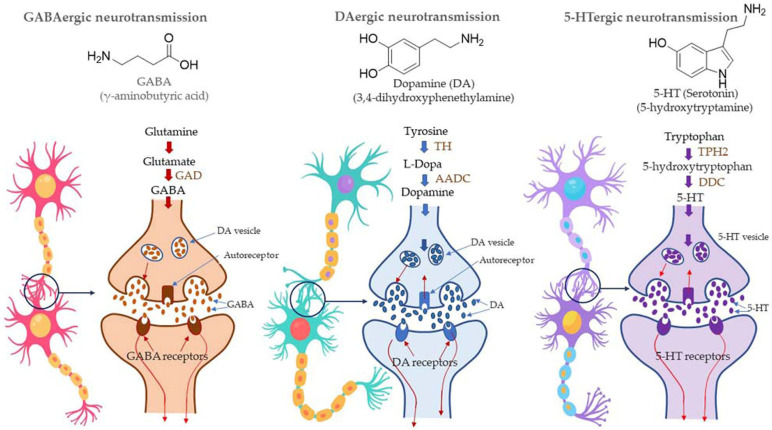
Syntheses of neurotransmitter molecules, viz., GABA (γ- Aminobutyric acid), dopamine, and serotonin (also called as 5-HT) and the mechanism of neurotransmission. AADC also known as DDC. Abbreviations; GAD (glutamate decarboxylase), TH (Tyrosine hydroxylase), AADC (aromatic amino acid decarboxylase), DDC (DOPA decarboxylase), TPH2 (s tryptophan hydroxylase 2).

**Figure 15:**
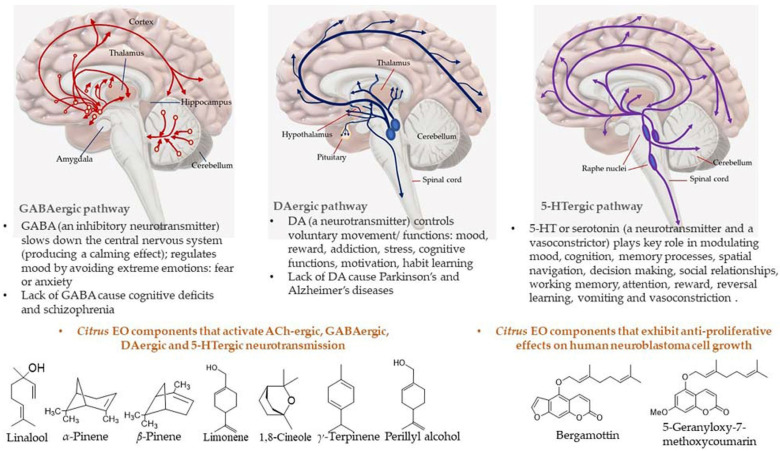
Neurotransmission pathways in GABAergic, DAergic, and 5-HTergic neurons and *Citrus* EO components that activate neurotransmission and exhibit anti-proliferative effects on human neuroblastoma cell growth.

Most of the drugs employed in the treatment for AD are synthesized chemically and have been observed to cause side effects, e.g., nausea or vomiting, hepatotoxicity, dyspepsia, myalgia, dizziness, anorexia, and so on. EOs have been observed to interact with a range of neurotransmitter pathways, namely noradrenergic (related to norepinephrine), 5-HTergic (related to serotonin), GABAergic (related to γ-aminobutyric acid), DAergic or dopaminergic (related to dopamine), etc. Furthermore, the specific compounds present in the EOs participate in specific action mechanisms, e.g., benzyl benzoate activates 5-HTergic and dopaminergic pathways and consequently exerts anxiolytic and anti-depressant effects [178]. Linalool and *β*-pinene interact with GABAergic pathway to produce similar effects. In this direction, other EO components, namely limonene benzyl alcohol has also been found to produce anxiolytic and anti-depressant effects. EOs can inhibit enzymes linked with hydrolysis of neurotransmitters, such as monoamine oxidase (MAO). Moreover, EOs possess antioxidative properties and can penetrate the blood–brain barrier. In this direction, Ademosun et al. carried out carried out inhibition assays of AChE and BChE, MAO, and lipid peroxidation [173]. The pathophysiological targets in diseased conditions of dementia, Alzheimer’s, and Parkinson’s are summarized in Figure 12. The Mechanism of action of citrus EOs to inhibit acetylcholinesterase (AChE), thereby increasing levels and duration of acetylcholine in the brain and assisting with cognition (learning and memory retention) is shown in Figure 13. The syntheses of different neurotransmitter molecules in the brain, namely GABA, dopamine, and serotonin, and the mechanism of neurotransmission are shown in Figure 14. The neurotransmission pathways in GABAergic, DAergic (dopaminergic), and 5-HTergic (serotoninergic) neurons and citrus EO components that activate neurotransmission and exhibit anti-proliferative effects on human neuroblastoma cell growth are shown in Figure 15.

The EO has been observed to inhibit AChE, BChE, and MAO in a dose-dependent manner. However, the EOs extracted from peels exhibited a significantly higher inhibition towards AChE compared with the EOs extracted from the seeds. On the other hand, the EOs from seeds exhibited a higher inhibition towards MAO activity compared with the peel EOs. Furthermore, the EOs also exhibited a decreasing effect on malondialdehyde (MDA) production which is present inside the brain homogenates. MAO activity is a crucial determinant in deactivating main neurotransmitters, such as serotonin, dopamine in the brain cells. This affects the overall behavior and mood of the patients suffering from Alzheimer’s disease. Zhou et al. [179] carried out passive avoidance test (PA) and open field habituation test (OFT) employing lemon EO components, *viz*., s-limonene and its derivatives-perillyl alcohol to investigate the effect of EOs on memory in rats. The rats were fed with s-limonene (100 mg/kg), s-perillyl alcohol (50 mg/kg) in their diets and scopolamine (1 mg/kg) was injected subcutaneously 30 min before the training test [179]. The lemon EO components showed a strong ability to improve learning and memory impaired by scopolamine in rats. BEO has been reported to exhibit antiproliferative activities in terms of inhibition against the survival and proliferation of SH-SY5Y neuroblastoma cells by activating multiple pathways resulting into necrosis and apoptotic cell death [69,180,181]. A summary of the studies on the application of citrus EOs in aromatherapy is presented in Table 1.

## 6. Summary

**Figure 16:**
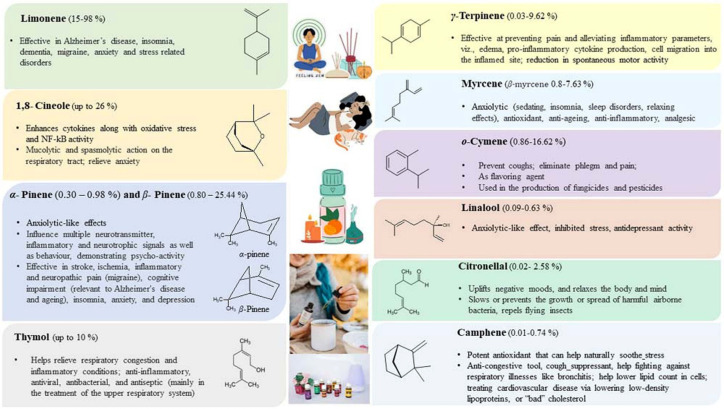
Therapeutic effects of majorly occurring component in citrus essential oil [202,208,209,210,211].

Citrus EOs are economical, eco-friendly, and natural alternatives to the synthetic compounds used in aromatherapy. Citrus-based EOs are mainly obtained from the leaves, flowers, and peels of young and ripened fruits, indirectly emphasizing waste management to save the environment from pollution and prevent contamination of the underground water table. Citrus EOs from waste peels used in aromatherapy help in relieving stress and stress-related disorders/diseases. The majorly occurring components present in citrus EOs and their therapeutic effects in aromatherapy have been summarized pictorially as below (Figure 16).

## Figures and Tables

**Table 1:** Pharmacological behavior of citrus EOs in aromatherapy.

*Citrus* Type	Particulars	In Vitro/In Vivo/Animal Model	Activity	References
**Bergamot orange** (*C. bergamia*) essential oil (CBEO)	Antioxidant behavior	In vivo model obtained from mouse hearts	Increase in transcription of genes involved in antioxidant responses Having lower IC_50_ O_2_^•−^ value in scavenging activity test than ascorbic acid and higher FRAP activity	[182]
	Mood disorder	BEO aromatherapy in alleviating depressive mood in postpartum women	Significantly improve the depressive mood Sleep quality was not significantly different	[103]
	Diseased condition	Acclimatization of the rats was performed	Relieve symptoms of stress-induced anxiety No overlapping between BEO and benzodiazepines behavioral effects Integrated effect on both 5-HT and GABA-A receptors	[183]
	Neuropharmacological studies	The elevated plus-maze and the hole-board tests were performed to study of BEO on rats	Usefulness in neuroprotection Chronic pain control Management of stress, anxiety, and anxiety-related conditions	[184]
	Antinociceptive effect	Effect of inhalation BEO on formalin-induced nociceptive response in mice.	Inhalation of BEO exerted antinociceptive activity. reduces formalin-induced licking/biting behavior. chronic pain relief in a stepwise therapeutic manner	[161]
**Sweet orange or navel orange** (*C. sinensis* L.) essential oil (CSEO)	Antioxidant behavior	Evaluated against the ROS-generating compound	Activity in DPPH assay was in a range of 6–23% for *C. sinensis* Decreased apoptosis in HaCat cells stimulated with H2O2. The levels of intracellular superoxide ion found to be lower	[185]
	Mood disorder	Aromatherapy during dental treatment	Lower degree of anxiety and a more cheerful attitude. To reduce salivary cortisol and pulse rate	[104]
	Physiological and psychological effect	Measurements were performed in a chamber with an artificial climate with 20 females	Significant decrease in oxyhemoglobin concentration in the right prefrontal cortex of the brain. Increases comfortable, relaxed, and natural feelings.	[104]
	Anxiolytic effect	Forty (40) male volunteers were allocated for the inhalation	Decreases the symptoms of anxiety Improves the mood	[186]
	Unpredictable mild stress	Randomized three-arm controlled trial	Significantly improved depression-like behaviors in CUMS mice by lowering sucrose preference, body weight, curiosity, and mobility Reducing immobility time and dyslipidemia	[17]
**Bitter orange** (*C. aurantium*) essential oil (CAEO)	Antioxidant behavior	DPPH scavenging test	contribute to the prevention of oxidation as antioxidants and free radical scavengers	[187]
	Mood disorder/anxiolytic effect	Collection of medullary material in patients with chronic myeloid leukemia (CML)	Anxiolytic effect and reduces the signs and symptoms associated with anxiety Decrease in the SBP and DBP	[188]
	Diseased condition/premenstrual syndrome (PMS)	Inhalation of 0.5 percent CAEO during the luteal phase of the menstrual cycle	Improved the symptoms of PMS Effective as a new and complementary therapeutic method for the emotions PMS symptoms in female.	[18]
	Sedative and hypnotic effects	Spielberger’s State-Trait Anxiety Inventory (STAI) was used after giving bitter orange flower powder capsule to post-menopausal women	Inhaling the CAEO greatly reduced anxiety	[189]
	Reduces pain	Study was a randomized clinical trial conducted with 126 eligible primiparous patients	Controls the enzymes in prostaglandins and reduces pain; controls the contractions caused by oxytocin and prostaglandins and exert anti-uterine pain effects	[190]
**Lemon** (*C. limon*) essential oil (CLEO)	Antioxidant behavior	DPPH radical scavenging assay	Lemon peel EO showed 55.09% inhibition of DPPH considerable antioxidant properties both in vitro and barley soup as food model	[191]
	Mood disorder/Anxiety	Thirty-nine sophomore nursing students (35 female and 4 males)	Positive effect on cognitive test anxiety	[192]
	Diseased condition/anxiolytic-like effect	Swiss mice model	Induce an anxiolytic behavior in mice no toxicity in vitro	[193]
	Treatment of dysmenorrhea	Population of this study amounted to 185	Psychological and physical benefits	[194]
	Effect on nausea among pregnant women	Control trial on 90 pregnant women	Effective in reducing pregnancy nausea and vomiting	[195]
**Mandarin** (*C. reticulata*) essential oil (CREO)	Antioxidant behavior	DPPH), 3-(N-morpholino) propane sulfonic acid (ABTS)	Exhibited moderate radical scavenging activity	[196]
	Mood disorder/mood and as a relaxing hypnotic agent	Frontal and parietal skulls of male Wistar rats implanted with electrodes for electroencephalographic (EEG)	CREO reduces REM sleep latency and enhanced the overall time and number of REM sleep episodes	[197]
	Anti-proliferative	Protective effects on bleomycin (BLM)-induced lung fibrosis in rats	Preventive effects on BLM-induced pulmonary fibrosis in rats Anti-proliferative effect against human embryonic lung fibroblasts	[198]
**Kaffir lime** (*C. hystrix*) essential oil (CHEO)	Antioxidant behavior	DPPH free radical scavenging assay	Potential antioxidant activity	[199]
	Stimulating effect	Forty healthy volunteers participated in the experiments	Reducing depression and stress in humans more alert, attentive, cheerful attitude	[200]
**Yuzu** (*C. junos*) essential oil (CJEO)	Antioxidant behavior	DPPH free radical scavenging test	Mature yuzu contains higher amounts of vitamin C and phenolics than other citrus fruits Significant dietary source of antioxidants	[201]
	Mood disorder	Inhaled administration (i.h.) of EOCJ for 90 min on mouse	Increased locomotor activity The anxiolytic-like effect	[202]
	Autonomic nervous system (ANS)	Study on seventeen women with subjective premenstrual symptoms	Therapeutic effects of yuzu fragrance on premenstrual symptoms (PMS) Can reduce premenstrual emotional symptoms Increased parasympathetic activity	[203]
	Physiological effect	Effect of 10-min inhalation of the yuzu scent on 21 women	Reduced heart rate (HR) and enhanced high-frequency power of heart rate variability (HRV), exhibiting parasympathetic nervous system activation, alleviation of negative emotional stress	[204]
	Human psychology	32 healthy participants enrolled in the study (16 men and 16 women, aged 20–24 years)	Oxyhemoglobin concentration in the prefrontal cortex increased Task performance improved after inhaling yuzu essential oil	[203]
**Neroli** (*C. aurantium*) essential oil (CAEO)	Antioxidant behavior	DPPH test	Prevention of oxidation as antioxidants and free radical scavengers. Essential oils in the old leaves had the maximum antioxidant activity	[43]
	Diseased condition/neurological disorder	Study on scopolamine-induced learning and memory deficit in rats	Repairing effects on memory and behavioral disorders Treatment of AD, insomnia, anxiety, and epilepsy	[205]
	Mood disorder/anxiolytic Effect	Study on patients with chronic myeloid leukemia (CML)	Diastolic pressure decreases Exhibits an anxiolytic effect and reduces the signs and symptoms associated with anxiety in patients with CML	[188]
	Antiseizure and anticonvulsant effect	Assessed in pentylenetetrazole (PTZ)-induced in mice	Anticonvulsant activity which supports the ethnomedicinal claims of the use of the plant in the management of seizure	[206]
	Effect on anxiety and perceived pain in women during labor	Study on 88 women during labor	Used as an alternative tool to relieve anxiety and perceived pain in women during all stages of labor	[207]

## Supplementary Materials

The following supporting information can be downloaded at: https://www.mdpi.com/article/10.3390/antiox11122374/s1, Figure S1: Climate sustainability and the annual production of citrus fruits in different geographical regions across the globe; Figure S2: Market segmentation of citrus essential oils; Figure S3: (a) Global citrus oil market by application, by the year 2018, (b) Citrus essential oil market value forecast (Citrus Oil Market by Product Type, 2022); Figure S4: Molecular structures of the volatile and non-volatile components present in Citrus EOs; Figure S5: Composition of EOs in different Citrus varieties; Table S1: Methods/Techniques of extracting Citrus essential oils; Table S2: Methods/techniques of characterization/authentication of Citrus essential oils. References [3,4,14,21,22,24,25,34,35,36,37,42,170,212,213,214,215,216,217,218,219] are cited in Supplementary Materials.
