# Supplementary material for: Citrus Essential Oils in Aromatherapy: Therapeutic Effects and Mechanisms
Source: Antioxidants (Basel). 2022 Nov 30;11(12):2374. doi: 10.3390/antiox11122374 (PMC9774566; doi:10.3390/antiox11122374)
Supplement: Supplementary file 1 [file antioxidants-11-02374-s001.zip › antioxidants-2021159-supplementary-v2.pdf]

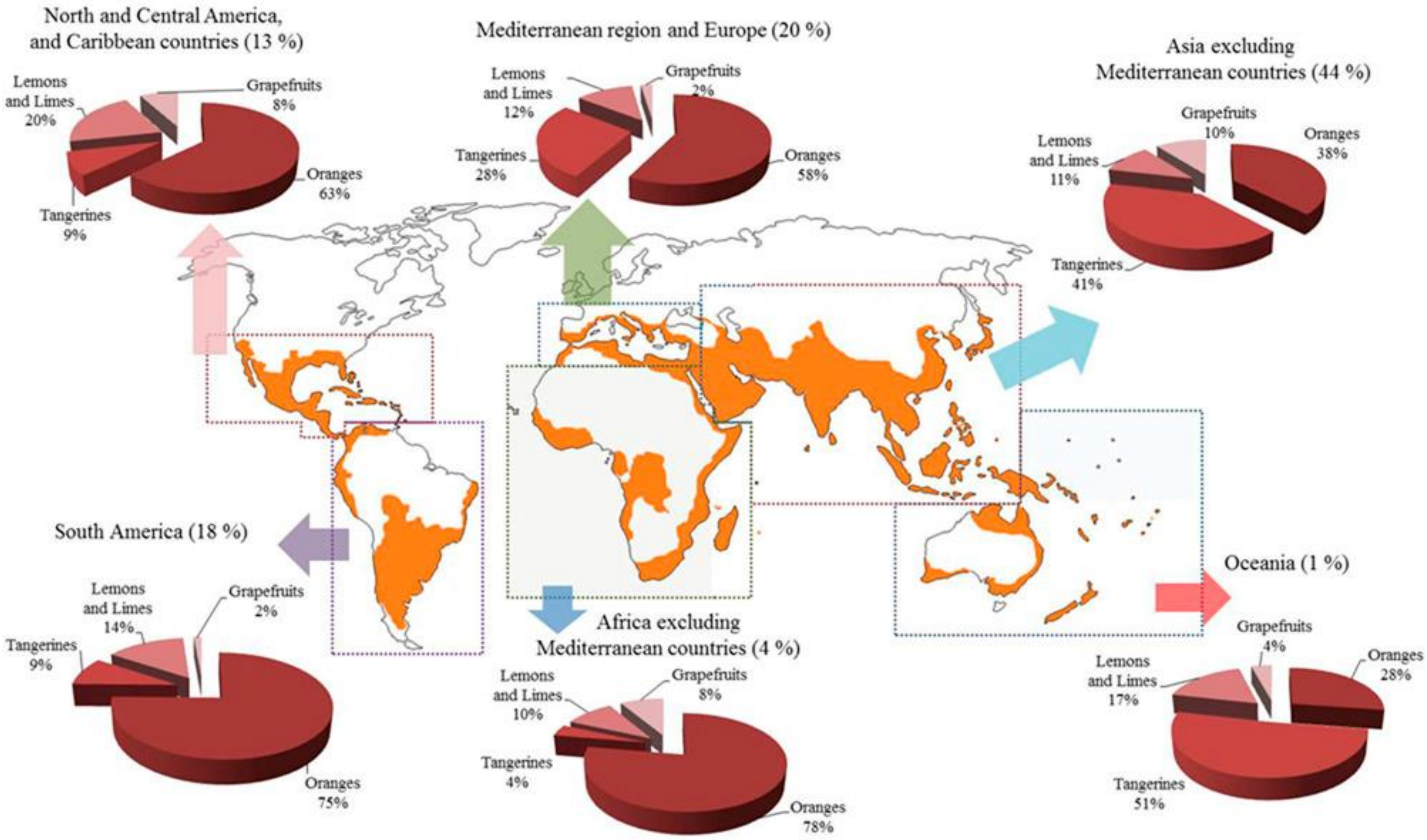

Figure S1: Climate sustainability and the annual production of citrus fruits in different geographical regions across the globe [212].

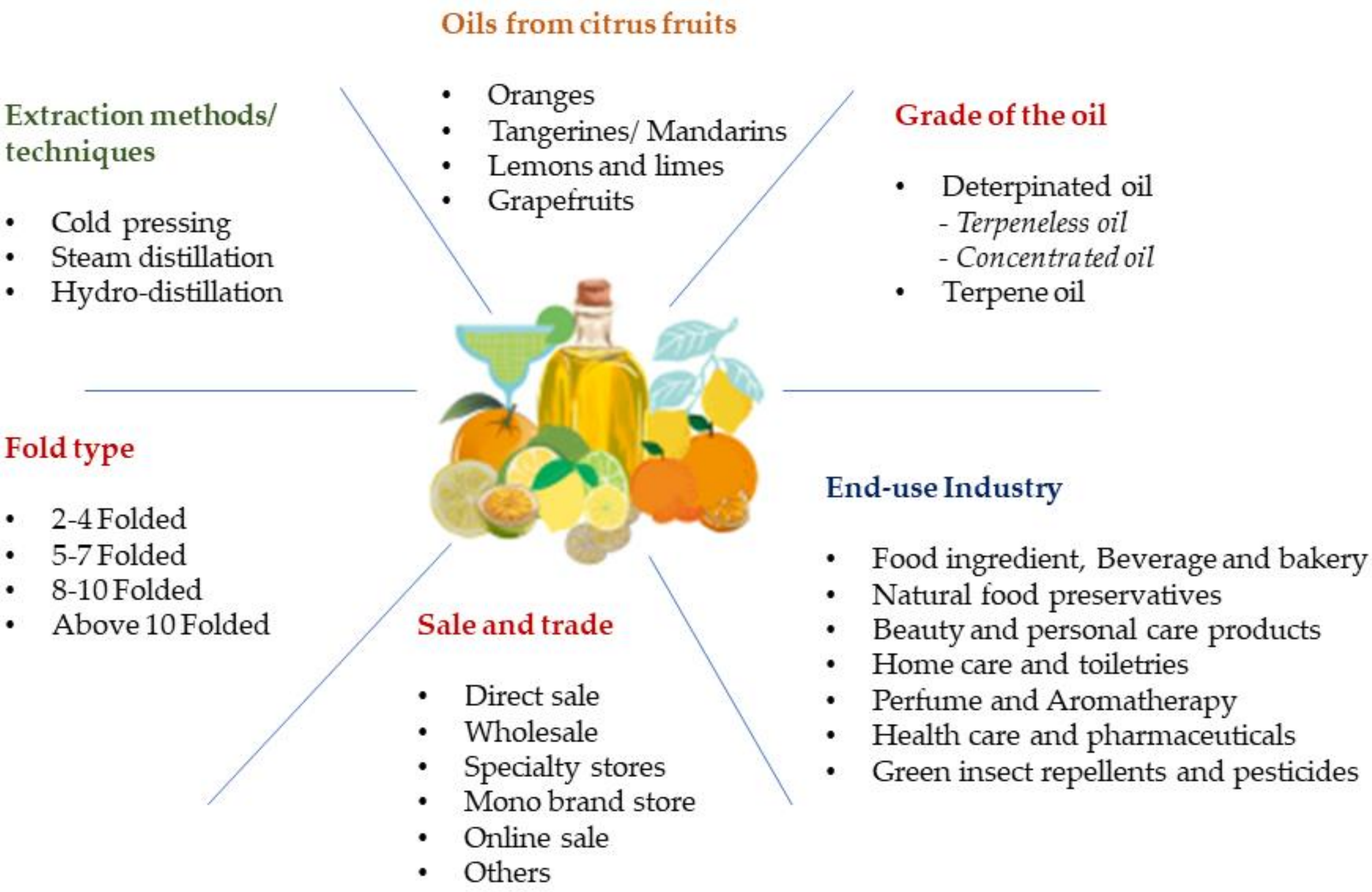

Figure S2: Market segmentation of citrus essential oils.

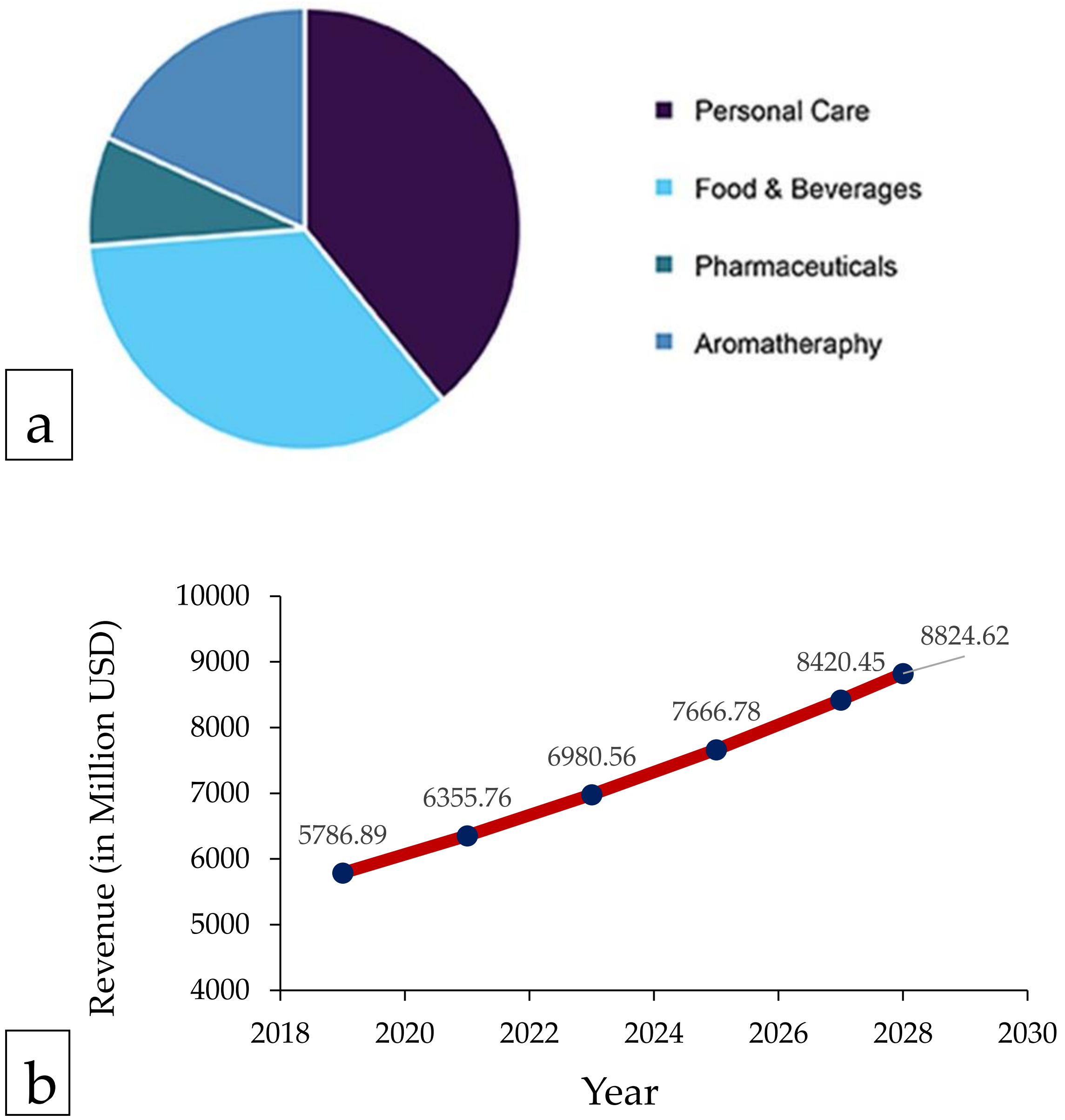

Figure S3 (a) Global citrus oil market by application, by the year 2018 [22,24]; **(b)** Citrus essential oil market value forecast (Citrus Oil Market by Product Type, 2022) [212].

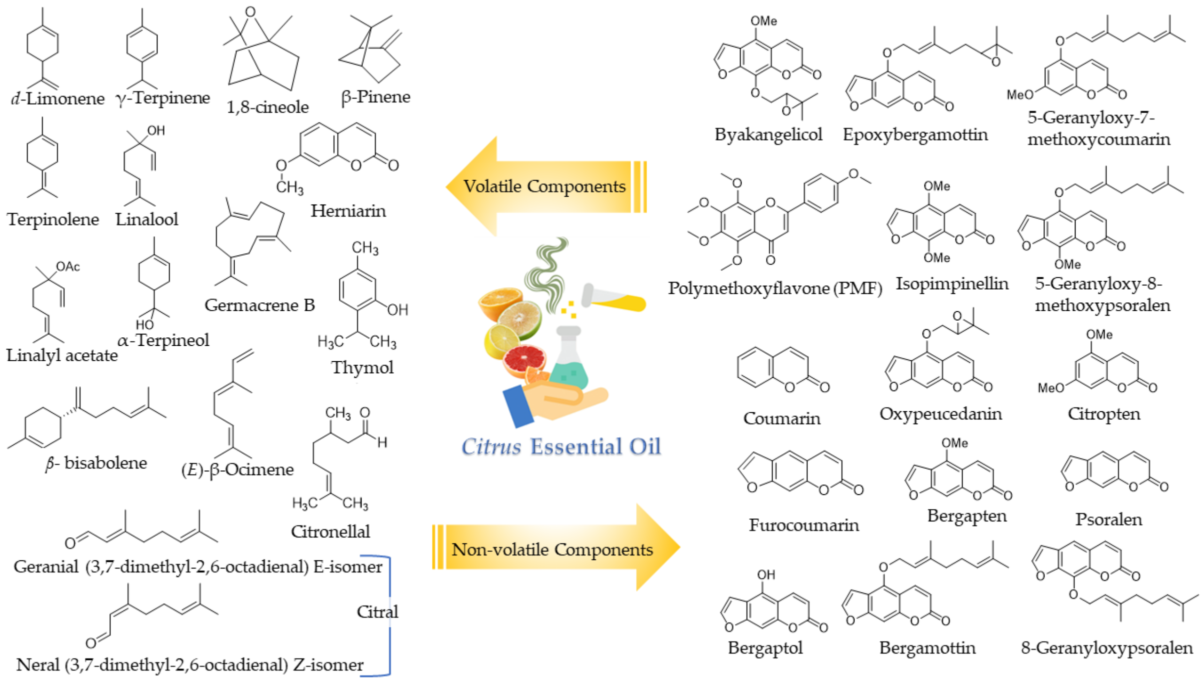

Figure S4. Molecular structures of the volatile and non-volatile components present in *Citrus* EOs.

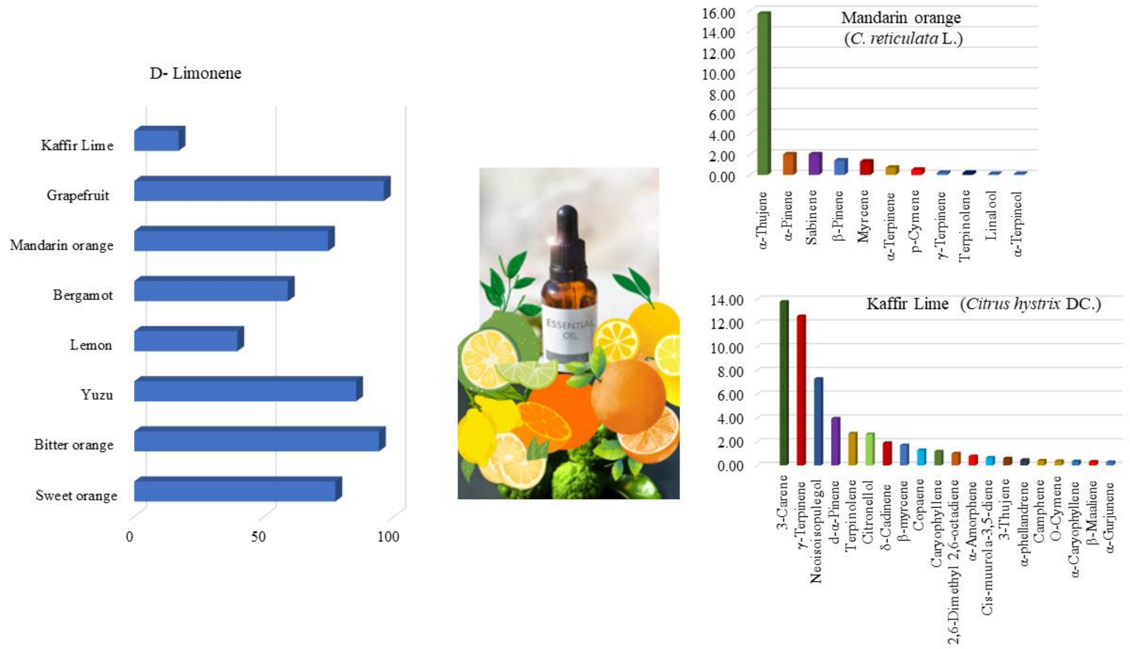

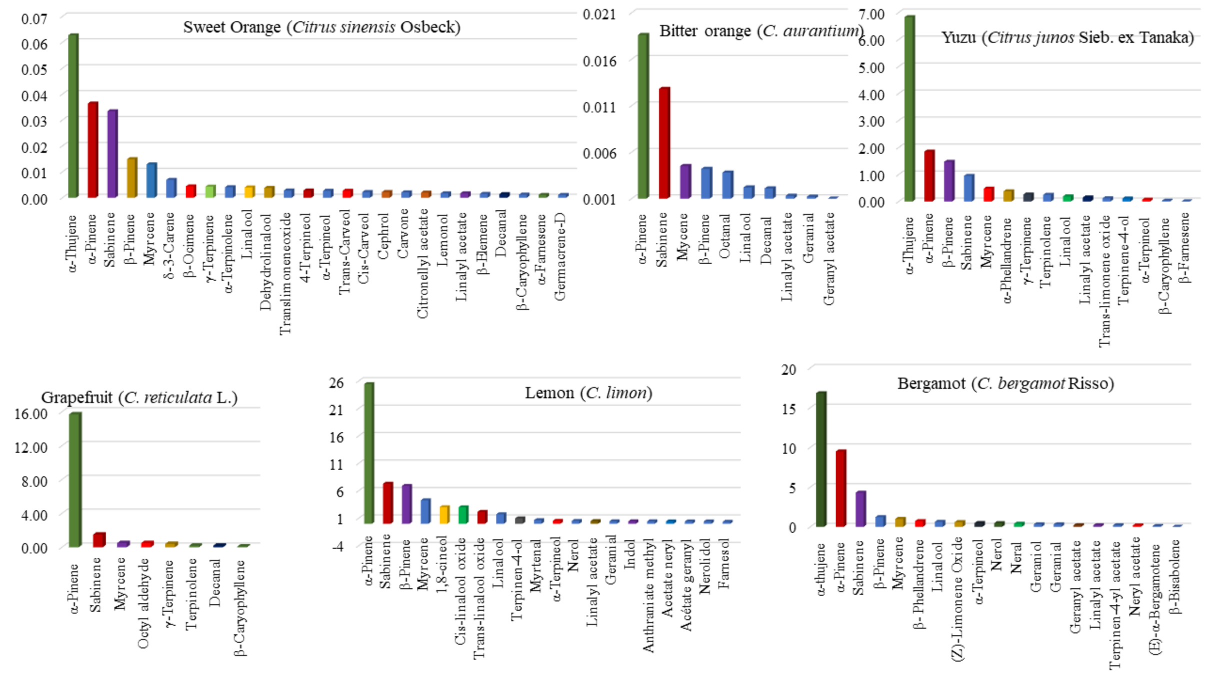

Figure S5. Composition of EOs in different *Citrus* varieties [3,14].

Table S1. Methods/ Techniques of extracting *Citrus* essential oils [4].

Hydro-distillation<br>Plant materials packed in a still compartment; water is added and brought to boil, or steam is directly injected into the plant sample; condensed mixture flows through condenser to a separator, where oil and bioactive compounds separate automatically from the water. Performed before dehydration of plant materials.
Cold Pressing<br>The peels are lacerated, across using mechanical pressure and oil flows; the oil is carried down to a decantation vessel in a stream of water; the emulsion collected and separated by centrifugation. The essential oil collected, dried over anhydrous sodium sulphate and stored at 4 °C.
Microwave Hydro-diffusion and Gravity<br>It combines microwave heating and gravity working at atmospheric pressure. The plant material is directly placed in a microwave reactor without solvent or water; heating is applied to rupture of the glands and cell receptacles; the extract diffuses outside the plant tissues; the extract drops under gravity out of the microwave reactor through the perforated Pyrex disc.
Microwave Steam (dry) Distillation<br>Fresh vegetable material is placed in a microwave reactor; internal heating of the water present in the plant material ruptures glands and oleiferous receptacles; the vapor passes through a condenser outside the microwave cavity; the distillate is collected continuously in a receiving flask.
Instant Controlled Pressure Drop Technique<br>The plant material is held under high pressure (0.08–1 MPa) for a short time (5–60 s); then the pressure is dropped instantly toward a vacuum (about 5 kPa); this results in auto evaporation of water, expansion, and rapid cooling of the product; the volatile material/ essential oil is extracted with in 1-4 min.
Steam Distillation<br>Food-grade solvents, viz., ethanol, benzene, dimethyl, or hexane are employed; plant material is treated with a low boiler solvent; the compounds transfer from one solvent to another owing to the difference in solubility between these two immiscible (or slightly soluble) solvents; the solvent is evaporated to obtain the desired compound.

Table S2. Methods/ techniques of characterization/authentication of *Citrus* essential oils.

Characterization/Authentication Technique	Particulars	References
Gas Chromatography-Mass Spectrometry (GC–MS)	Chemical composition and principal constituents of the citrus essential oil	[170]
Multidimensional Gas Chromatography – Flame Ionization Detection (MDGC-GCFID)	Determination of enantiomeric composition in Lemon peel	[213]
Multidimensional Gas Chromatography – Flame Ionization Detection – Selected Ion Monitoring (MDGC-GC-MSSIM)	Determination of enantiomeric excess of R-(C)-limonene in Lemon peel oil (97.1 and 97.4%)	
Enantioselective Gas Chromatography (esGC) and Multidimensional Gas Chromatography (MDGC)	Enantiomeric composition and distribution in different *Citrus* EOs	[42]
Simultaneous Distillation and Extraction (SDE)-GC-MS and MDGC-MS	Analysis of volatile flavor compounds of yuzu, lemon, and lime; Enantiomeric ratios of chiral and authentication of quality	[34]
Supercritical Fluid Extraction GC–MS (SFE GC–MS) Involving Use of Multidimensional GC	Extraction and determination of composition; separation of enantiomers	[214]
Enantioselective capillary gas chromatography (enantio-cGC) and comparative isotope ratio mass spectrometry (IRMS), coupled on-line with conventional GC	Authenticity control of flavors and EOs	[215]
Enantioselective Capillary Gas Chromatography and Isotope Ratio Mass Spectrometry, Coupled Online with Capillary Gas Chromatography on an HP5 Column	Analysis and authenticity investigations of lemon (*Citrus limon*) EOs	[216]
Online Gas Chromatography Pyrolysis Isotope Ratio Mass Spectrometry (HRGC–P–IRMS)	Authentication of the flavor compounds	[217]
Isotope Ratio Mass Spectrometry Online Coupled with capillary Gas Chromatography (GC-Py-IRMS)	Authentication and genuineness of mandarin *Citrus reticulata* Blanco EOs	[218]
Enantioselective Gas Chromatography in Flavor and Fragrance Analysis	Authentication of fragrance compounds	[219]
Headspace–Solid Phase Microextraction Coupled to GC– C–IRMS	Authentication of *Citrus* EOs	[36]

Multi-Dimensional Gas Chromatography (MDGC) and GCC-IRMS	Authentication of Neroli and lime oils and determine the level of adulteration	[35]
Ultra-High Performance Liquid Chromatography–Time-of-Flight–Mass Spectrometry (UHPLC– TOFMS)	Fingerprinting for Lemon Oil	[37]
ATR (attenuated total reflectance)/FTIR as well as NIR-FT Raman spectroscopy	Determination of composition of *Citrus* oils	[220]
